# Dietary Approaches to Obstructive Sleep Apnea: Translating Mechanistic Pathways into Clinical Practice

**DOI:** 10.3390/clockssleep8040058

**Published:** 2026-09-22

**Authors:** Tatiana Palotta Minari, José Fernando Vilela-Martin, Luciana Pellegrini Pisani

**Affiliations:** 1 Department of Bioscience, Federal University of São Paulo (UNIFESP), Santos 11015-020, SP, Brazil; pisani@unifesp.br; 2 Department of Internal Medicine, Medical School São José do Rio Preto, FAMERP, São José do Rio Preto 15090-000, SP, Brazil; vilelamartin@uol.com.br

**Keywords:** obstructive sleep apnea, nutrition, precision nutrition, Mediterranean diet, gut microbiota, obesity, GLP-1 receptor agonists, inflammation, cardiovascular disease

## Abstract

Obstructive sleep apnea (OSA) is a highly prevalent and heterogeneous sleep disorder characterized by recurrent upper airway collapse during sleep, resulting in intermittent hypoxia, sleep fragmentation, sympathetic activation, and increased cardiometabolic risk. Although obesity is the most recognized modifiable risk factor, growing evidence indicates that nutritional factors influence OSA through mechanisms extending beyond body weight regulation, including systemic inflammation, oxidative stress, endothelial dysfunction, gut microbiota alterations, and metabolic dysregulation. The emergence of precision medicine and incretin-based therapies has further highlighted the need to integrate personalized nutritional approaches into OSA management. A literature search was conducted in PubMed/MEDLINE, Scopus, and Web of Science to identify experimental studies, observational studies, randomized clinical trials, systematic reviews, meta-analyses, and international clinical guidelines addressing nutritional mechanisms, dietary patterns, gut microbiota, chrononutrition, incretin-based therapies, and precision nutrition in adults with OSA. Current evidence suggests that nutritional interventions may influence OSA through multiple biological pathways beyond weight reduction. Mediterranean and DASH dietary patterns are associated with favorable cardiometabolic effects, while dietary fiber, polyphenols, omega-3 fatty acids, and other antioxidant compounds may modulate pathways related to inflammation, oxidative stress, endothelial function, and metabolic regulation. However, evidence directly demonstrating improvements in objective respiratory outcomes remains limited and heterogeneous. Emerging research also suggests potential roles for gut microbiota modulation and chrononutrition in metabolic and sleep regulation, although most evidence remains mechanistic, observational, or indirect. In addition, glucagon-like peptide-1 receptor agonists and dual incretin agonists have demonstrated clinically relevant reductions in body weight and OSA severity, providing opportunities for integration with nutritional management. Nevertheless, evidence supporting phenotype-specific and biomarker-guided nutritional interventions remains insufficient. Nutrition represents a promising adjunctive strategy in the multidisciplinary management of OSA by targeting inflammatory, metabolic, vascular, and microbiome-related mechanisms that extend beyond weight loss alone. Future research should focus on precision nutrition approaches integrating metabolic phenotyping, biomarkers, gut microbiota, and digital health technologies to optimize personalized treatment and improve long-term clinical outcomes.

## 1. Introduction

Obstructive sleep apnea (OSA) is one of the most prevalent chronic sleep disorders worldwide, affecting nearly one billion adults aged 30–69 years and representing a major public health challenge because of its substantial cardiovascular, metabolic, neurocognitive, and socioeconomic consequences [1,2]. OSA is characterized by recurrent episodes of partial or complete upper airway obstruction during sleep, leading to intermittent hypoxia, hypercapnia, sleep fragmentation, large intrathoracic pressure swings, and sympathetic nervous system activation [1]. These physiological disturbances trigger a cascade of molecular and systemic alterations, including oxidative stress, chronic low-grade inflammation, endothelial dysfunction, autonomic imbalance, and metabolic dysregulation, that contribute to hypertension, coronary artery disease, atrial fibrillation, heart failure, stroke, type 2 diabetes mellitus, metabolic dysfunction-associated steatotic liver disease (MASLD), and increased all-cause mortality [3].

Obesity remains the strongest modifiable risk factor for OSA, with approximately 60–70% of patients presenting overweight or obesity [4,5]. Excess visceral, cervical, and peripharyngeal adiposity contributes to upper airway narrowing, increased pharyngeal collapsibility, reduced lung volumes, and impaired respiratory mechanics [3,6,7]. Nevertheless, obesity alone does not fully explain the remarkable clinical heterogeneity observed among patients with OSA [1,5]. Some individuals with severe obesity do not develop clinically significant OSA, whereas many lean individuals exhibit moderate-to-severe disease, indicating that anatomical susceptibility interacts with non-anatomical mechanisms such as ventilatory control instability, impaired upper airway muscle responsiveness, low arousal threshold, fluid redistribution, and metabolic dysfunction [1,5,8,9]. Consequently, OSA is increasingly recognized as a heterogeneous disorder comprising multiple physiological phenotypes and endotypes that influence disease severity, cardiometabolic risk, and therapeutic responsiveness [10,11].

Continuous positive airway pressure (CPAP) remains the first-line therapy for moderate-to-severe OSA; however, long-term adherence remains suboptimal, and CPAP alone does not reverse obesity, insulin resistance, systemic inflammation, endothelial dysfunction, or residual cardiovascular risk [10,12,13,14,15]. These limitations have stimulated growing interest in complementary therapeutic strategies capable of addressing the systemic mechanisms underlying OSA [16,17,18]. Among these, nutritional interventions have emerged as promising non-pharmacological approaches because dietary factors influence not only body weight but also inflammation, oxidative stress, endothelial function, autonomic regulation, insulin sensitivity, skeletal muscle metabolism, gut microbiota composition, and circadian biology [2,5,18]. Moreover, the recent introduction of glucagon-like peptide-1 receptor agonists (GLP-1RAs) and dual incretin agonists has renewed interest in integrating nutritional management with pharmacological therapies to achieve greater improvements in both metabolic health and OSA severity [1,10,16].

Despite these advances, current nutritional recommendations for patients with OSA remain largely extrapolated from obesity and cardiovascular disease guidelines rather than being specifically tailored to the heterogeneous pathophysiology of OSA [5]. Furthermore, most published reviews have primarily focused on weight loss, bariatric surgery, or isolated dietary interventions, with limited discussion of the molecular mechanisms linking nutrition to OSA pathogenesis [2,19,20,21,22,23,24,25,26,27,28]. Emerging concepts, including precision nutrition, immunometabolism, gut microbiota, chrononutrition, metabolomics, and the integration of dietary strategies with incretin-based therapies, have received comparatively little attention, despite their potential to transform individualized OSA management [2,5]. Therefore, an updated synthesis integrating these rapidly evolving fields within the context of precision medicine is warranted.

This narrative review aims to critically examine current evidence regarding the role of nutrition in the pathophysiology and management of OSA, emphasizing mechanisms beyond weight loss, including inflammation, oxidative stress, endothelial dysfunction, gut microbiota, chrononutrition, and metabolic regulation. In addition, we discuss emerging evidence supporting precision nutrition and its integration with incretin-based therapies and other personalized therapeutic approaches, while identifying current knowledge gaps and future research priorities for individualized nutritional management of patients with OSA.

## 2. Methodology

### 2.1. Study Design and Purpose

This narrative review was developed to critically synthesize current evidence on the role of nutrition in the pathophysiology and management of obstructive sleep apnea (OSA). The review emphasizes mechanisms beyond weight loss and integrates emerging concepts in precision nutrition, chronobiology, gut microbiota, and incretin-based therapies.

### 2.2. Databases and Search Period

A comprehensive literature search was conducted in PubMed/MEDLINE, Scopus, and Web of Science. The search covered publications from January 2000 to June 2026, ensuring inclusion of both foundational and contemporary studies relevant to nutrition, metabolism, chronobiology, gut microbiota, and precision medicine in OSA.

### 2.3. Search Strategy

The search strategy combined controlled vocabulary and free-text terms. Boolean operators were applied to structure the search, with “AND” linking major concepts and “OR” grouping synonyms within each concept. Terms related to OSA (“obstructive sleep apnea”, “OSA”, “sleep-disordered breathing”) were combined with terms related to nutrition (“nutrition”, “diet”, “dietary patterns”, “Mediterranean diet”, “DASH diet”, “chrononutrition”), biological mechanisms (“inflammation”, “oxidative stress”, “endothelial dysfunction”, “insulin resistance”, “gut microbiota”), and precision medicine or incretin-based therapies (“precision nutrition”, “GLP-1 receptor agonists”, “dual incretin agonists”). Equivalent search structures were adapted for Scopus and Web of Science. As stated in the manuscript, “A literature search was conducted in PubMed/MEDLINE, Scopus, and Web of Science to identify experimental studies, observational studies, randomized clinical trials, systematic reviews, meta-analyses, and international clinical guidelines. Complete database-specific search strings are provided in Supplementary Materials.

### 2.4. Eligibility Criteria

Studies were eligible for inclusion if they involved adults aged 18 years or older with OSA diagnosed by polysomnography or validated methods, and if they addressed nutritional mechanisms, dietary interventions, metabolic regulation, gut microbiota, chrononutrition, or precision nutrition. Randomized controlled trials, observational studies, experimental investigations, systematic reviews, meta-analyses, and international clinical guidelines were considered. Articles were included if written in English, Portuguese, or Spanish. Pediatric studies were excluded unless they provided explicit relevance to adult populations, and articles lacking methodological detail or containing only opinion-based content were not included.

### 2.5. Study Selection Process

Study selection occurred in two stages. First, two reviewers (TPM and LPP) independently screened titles and abstracts retrieved from the database searches. Articles deemed potentially relevant were then evaluated in full text. Disagreements were resolved through discussion, and when necessary, a third reviewer (JFVM) provided adjudication. As stated in the manuscript.

The database search identified 200 records, including 82 records from PubMed/MEDLINE, 67 from Scopus, and 51 from Web of Science Core Collection. After removal of 31 duplicate records, 169 records remained for title and abstract screening. Following screening, 132 articles were assessed in full text, of which 13 were excluded according to the predefined eligibility criteria. A total of 119 publications were included in the final narrative synthesis.

### 2.6. Evidence Appraisal

Because this is a narrative review, no formal risk-of-bias assessment or quantitative meta-analysis was performed. Instead, a structured qualitative appraisal was applied. Priority was given to OSA-specific randomized controlled trials, systematic reviews, meta-analyses, and international guidelines. Evidence was interpreted according to its nature: direct evidence from OSA studies reporting respiratory outcomes such as AHI and ODI; indirect evidence from populations with obesity, metabolic syndrome, or cardiovascular disease; and mechanistic or preclinical evidence from experimental models, microbiota analyses, and chronobiology research.

### 2.7. Data Synthesis

The synthesis of findings was organized into thematic domains, including biological mechanisms linking nutrition to OSA, dietary patterns, specific nutrients and bioactive compounds, incretin-based therapies, and precision nutrition approaches involving phenotypic and endotypic stratification. The analysis emphasized mechanistic pathways, clinical evidence, gaps in knowledge, and implications for individualized nutritional management of OSA.

## 3. Nutritional Mechanisms in the Pathophysiology of Obstructive Sleep Apnea

OSA is increasingly recognized as a multifactorial disorder resulting from complex interactions among anatomical susceptibility, neuromuscular dysfunction, ventilatory control instability, arousal threshold, and metabolic abnormalities [10,26,29,30,31,32]. Although obesity remains the most important modifiable risk factor, accumulating evidence indicates that the pathophysiological relationship between nutrition and OSA extends far beyond excess adiposity [5,33,34,35,36,37]. Dietary factors influence numerous biological processes implicated in OSA development and progression, including adipose tissue distribution, systemic inflammation, oxidative stress, endothelial dysfunction, insulin resistance, autonomic regulation, circadian biology, skeletal muscle function, and gut microbiota composition [2,5,35,38,39]. Consequently, nutritional interventions should be viewed not merely as weight-loss strategies but as modulators of multiple interconnected pathways that contribute to disease severity and cardiometabolic complications [5,39,40,41,42,43] (Figure 1).

### 3.1. Obesity, Body Fat Distribution, and Upper Airway Anatomy

Obesity contributes to OSA through both mechanical and metabolic mechanisms. Rather than total body weight alone, the regional distribution of adipose tissue appears to be a major determinant of upper airway collapsibility [44,45,46]. Fat accumulation around the neck, tongue, soft palate, and parapharyngeal tissues reduces the caliber of the upper airway and increases extraluminal tissue pressure, thereby predisposing to pharyngeal collapse during sleep [44,45]. Imaging studies using magnetic resonance imaging (MRI) and computed tomography have demonstrated that tongue fat volume is significantly greater in patients with OSA than in weight-matched controls without sleep-disordered breathing, suggesting that ectopic fat deposition directly contributes to airway obstruction [32,39,44,45].

Visceral adiposity also exerts indirect effects on respiratory physiology [45,46,47,48,49]. Increased abdominal fat reduces functional residual capacity and expiratory reserve volume, decreasing caudal traction on the upper airway and promoting pharyngeal instability [50,51,52,53]. Furthermore, obesity-related reductions in lung volume impair upper airway dilator muscle efficiency, increasing the propensity for airway collapse, particularly during rapid eye movement (REM) sleep [54,55].

Importantly, not all individuals with obesity develop OSA, and disease severity is often poorly correlated with body mass index (BMI) alone [55,56,57]. These observations underscore the importance of considering body composition, ectopic fat accumulation, craniofacial anatomy, and metabolic phenotype rather than relying exclusively on BMI when evaluating nutritional risk in OSA [5]. Beyond anatomical alterations, adipose tissue functions as an active endocrine organ that secretes adipokines, cytokines, and inflammatory mediators capable of influencing respiratory control and systemic metabolism [3]. Consequently, the detrimental effects of obesity on OSA extend well beyond mechanical airway narrowing [3,58,59,60].

### 3.2. Chronic Low-Grade Inflammation

Persistent low-grade systemic inflammation is one of the hallmark biological features linking obesity, metabolic dysfunction, and OSA [61,62,63]. Recurrent cycles of intermittent hypoxia activate hypoxia-inducible factor-1α (HIF-1α) and nuclear factor kappa B (NF-κB), leading to increased transcription of numerous pro-inflammatory genes [37,58]. Consequently, circulating concentrations of tumor necrosis factor-alpha (TNF-α), interleukin-6 (IL-6), interleukin-1β (IL-1β), monocyte chemoattractant protein-1 (MCP-1), and C-reactive protein (CRP) are elevated in patients with moderate-to-severe OSA [53].

Adipose tissue further amplifies this inflammatory milieu through macrophage infiltration and polarization toward a pro-inflammatory M1 phenotype [8,18,34]. These activated immune cells produce cytokines that impair insulin signaling, endothelial function, and vascular homeostasis, thereby increasing cardiovascular risk [8]. Importantly, inflammation itself may contribute to upper airway dysfunction by promoting neuromuscular impairment, tissue edema, and altered pharyngeal muscle contractility, creating a vicious cycle between airway obstruction and inflammatory activation [14,49,51].

Diet strongly modulates systemic inflammatory responses [56,57,58,59]. Western dietary patterns characterized by excessive consumption of ultra-processed foods, saturated fats, refined carbohydrates, and added sugars promote activation of inflammatory signaling pathways, whereas Mediterranean-style dietary patterns rich in fruits, vegetables, legumes, whole grains, olive oil, nuts, and fish may be associated with lower circulating inflammatory biomarkers [2,52,54]. Therefore, nutritional interventions and other lifestyle changes targeting inflammatory pathways may represent an important adjunctive strategy for reducing both OSA severity and its cardiometabolic consequences [5,55,64,65,66,67,68,69,70].

### 3.3. Oxidative Stress and Endothelial Dysfunction

Intermittent hypoxia represents one of the defining pathophysiological characteristics of OSA and constitutes a potent inducer of oxidative stress [58]. Repeated cycles of hypoxia and reoxygenation resemble ischemia–reperfusion injury, resulting in excessive production of reactive oxygen species (ROS) by mitochondria, NADPH oxidase, xanthine oxidase, and uncoupled endothelial nitric oxide synthase [25,26,27,28,48,49,58,68].

Excessive ROS generation overwhelms endogenous antioxidant defenses, leading to lipid peroxidation, protein oxidation, DNA damage, mitochondrial dysfunction, and activation of redox-sensitive inflammatory pathways [26,53,54,58]. Oxidative stress also reduces nitric oxide bioavailability through direct inactivation and promotes endothelial dysfunction, vascular stiffness, and impaired vasodilation, mechanisms strongly associated with hypertension and cardiovascular disease in patients with OSA [27,55,56,58].

Nutritional factors substantially influence oxidative balance [2,52]. Diets abundant in antioxidant compounds, including vitamins C and E, carotenoids, selenium, polyphenols, and flavonoids, have been associated with improved endothelial function and reduced oxidative damage in cardiometabolic diseases [2,4,54,55,56,57]. Polyphenol-rich foods such as berries, extra-virgin olive oil, cocoa, green tea, and pomegranate exhibit anti-inflammatory and antioxidant properties that may attenuate ROS-mediated vascular injury [2,4,56,57]. Although evidence specifically addressing antioxidant supplementation in OSA remains limited, current findings support dietary patterns emphasizing naturally antioxidant-rich foods rather than isolated micronutrient supplementation [4].

### 3.4. Insulin Resistance and Metabolic Dysfunction

The relationship between OSA and insulin resistance is bidirectional and independent of obesity in many individuals [9,23,32,59]. Intermittent hypoxia impairs glucose metabolism through activation of sympathetic pathways, increased hepatic gluconeogenesis, pancreatic β-cell dysfunction, and reduced skeletal muscle glucose uptake [58,60]. Simultaneously, obesity-associated inflammation exacerbates insulin resistance by disrupting insulin receptor signaling via serine phosphorylation of insulin receptor substrate proteins [35,62,63,64].

Patients with OSA exhibit a higher prevalence of metabolic syndrome, type 2 diabetes mellitus, dyslipidemia, and metabolic dysfunction-associated MASLD compared with the general population [60,71,72,73,74]. These metabolic abnormalities further worsen airway function by promoting ectopic fat deposition and systemic inflammation, reinforcing a self-perpetuating cycle between metabolic dysfunction and sleep-disordered breathing [68,69].

Nutritional interventions capable of improving insulin sensitivity, including Mediterranean dietary patterns, increased dietary fiber intake, reduced consumption of refined carbohydrates, and improved dietary quality, may therefore provide benefits extending beyond body weight reduction alone [2,67,75,76,77]. Recent clinical trials evaluating GLP-1RAs and dual incretin agonists further reinforce the central role of metabolic regulation in OSA management by demonstrating clinically meaningful reductions in both body weight and apnea–hypopnea index (AHI), although the relative contribution of weight-dependent versus weight-independent mechanisms remains under investigation [1,16,33].

### 3.5. Adipokines and Neuroendocrine Regulation

Adipose tissue-derived hormones play an important role in linking nutritional status with respiratory physiology [1,78]. Leptin, traditionally recognized for regulating appetite and energy expenditure, also stimulates central respiratory drive [1]. However, obesity is characterized by leptin resistance, resulting in impaired ventilatory stimulation despite elevated circulating leptin concentrations [33,64,78]. This phenomenon may contribute to ventilatory instability and reduced responsiveness to hypercapnia in patients with OSA [64,78].

Conversely, adiponectin, an adipokine with potent anti-inflammatory and insulin-sensitizing properties, may be reduced in obesity and OSA [64,68]. Lower adiponectin concentrations are associated with endothelial dysfunction, increased oxidative stress, and higher cardiovascular risk [64]. Nutritional interventions that promote weight reduction and improve metabolic health generally increase adiponectin levels while reducing leptin resistance, suggesting another mechanism through which diet may influence OSA pathophysiology [33,67,77].

Collectively, these mechanisms demonstrate that nutrition influences OSA through multiple interconnected pathways that extend substantially beyond reductions in body weight [79,80,81,82,83,84,85]. Dietary modulation of inflammation, oxidative stress, endothelial function, insulin resistance, adipokine secretion, and ectopic fat accumulation provides a strong biological rationale for incorporating nutritional interventions into comprehensive OSA management [83]. Nevertheless, these interactions are bidirectional, as OSA itself profoundly alters appetite regulation, food preferences, energy metabolism, circadian rhythms, and epigenetic processes [66,84]. Understanding this reciprocal relationship is essential for developing effective personalized nutritional strategies and is discussed in the following section (Table 1).

## 4. The Bidirectional Relationship Between Nutrition and Obstructive Sleep Apnea

The relationship between nutrition and OSA is complex and bidirectional. While obesity and poor dietary habits contribute to the development and progression of OSA, the disorder itself induces profound metabolic, hormonal, behavioral, and circadian alterations that negatively influence eating behavior, dietary quality, and energy homeostasis [55,56,57,58,59,60,61,62,63]. Consequently, many patients enter a self-perpetuating cycle in which sleep disruption promotes adverse metabolic adaptations that favor weight gain and unhealthy food choices, ultimately exacerbating OSA severity and its cardiometabolic consequences [55].

### 4.1. Sleep Fragmentation and Appetite Dysregulation

Sleep fragmentation, one of the hallmarks of OSA, disrupts neuroendocrine pathways involved in appetite regulation. Recurrent arousals activate the hypothalamic–pituitary–adrenal (HPA) axis and increase sympathetic nervous system activity, leading to alterations in the secretion of hormones that regulate hunger and satiety [86]. Studies have demonstrated that inadequate sleep is associated with increased circulating ghrelin concentrations, reduced leptin signaling, elevated cortisol levels, and impaired insulin sensitivity, creating a hormonal environment that promotes positive energy balance [6,58,73,74].

Although leptin concentrations are frequently elevated in patients with obesity and OSA, this increase reflects leptin resistance rather than enhanced satiety signaling [75]. Impaired leptin sensitivity reduces hypothalamic responsiveness, leading to persistent hunger despite adequate or excessive energy stores. Simultaneously, elevated ghrelin stimulates appetite, particularly for energy-dense foods rich in refined carbohydrates and saturated fats [51]. These hormonal disturbances may partly explain why patients with untreated OSA often report increased caloric intake and greater difficulty adhering to dietary interventions [53].

Sleep fragmentation also influences the secretion of glucagon-like peptide-1 (GLP-1), peptide YY (PYY), and other gut-derived hormones involved in appetite regulation, although current evidence remains inconsistent [1,33,77]. Further research is needed to clarify whether restoring sleep continuity through OSA treatment normalizes these hormonal responses independently of weight loss [1,77]. Disrupted sleep architecture alters multiple neuroendocrine pathways involved in appetite regulation. Inadequate sleep is associated with increased circulating ghrelin concentrations, reduced leptin signaling, elevated cortisol levels, and impaired insulin sensitivity [19]. In addition to these hormones, orexin (hypocretin) plays a central role in linking metabolic status with wakefulness and feeding behavior. Orexin neurons promote arousal, increase respiratory drive, and stimulate appetite, and their activity is strongly influenced by nutritional cues such as glucose availability, amino acid intake, and circulating ghrelin and leptin levels [1,6,33,77].

Disrupted sleep in OSA may lead to dysregulated orexin signaling, increasing daytime sleepiness, promoting cravings for energy-dense foods, and destabilizing sleep–wake transitions [1]. High-fat diets, irregular meal timing, and nocturnal eating have been shown to increase orexin activity, potentially exacerbating arousal frequency and ventilatory instability [58]. Conversely, balanced dietary patterns and regular feeding schedules may help stabilize orexin signaling, supporting both appetite regulation and sleep continuity in patients with OSA [54].

### 4.2. Food Preferences and Eating Behavior

OSA not only alters physiological appetite regulation but also affects food-related decision-making and eating behavior. Studies have shown that sleep deprivation enhances activation of reward-related brain regions, including the nucleus accumbens, orbitofrontal cortex, amygdala, and insula, while simultaneously reducing activity within the prefrontal cortex, an area responsible for executive function and inhibitory control [51]. As a result, individuals experiencing chronic sleep disruption exhibit increased preference for highly palatable foods characterized by high sugar, sodium, and saturated fat content [50,51].

Observational studies report lower adherence to healthy dietary patterns among individuals with OSA, including reduced consumption of fruits, vegetables, legumes, and whole grains, together with greater intake of ultra-processed foods, sugar-sweetened beverages, and fast foods [2,54]. Excessive daytime sleepiness may further contribute to irregular meal timing, increased snacking frequency, emotional eating, and reduced motivation to prepare healthy meals or engage in regular physical activity [40,82].

Moreover, fatigue and impaired cognitive performance associated with untreated OSA may decrease adherence to lifestyle interventions, thereby limiting the effectiveness of conventional weight-management programs. These behavioral factors reinforce the importance of integrating nutritional counseling with behavioral and sleep-focused interventions in multidisciplinary OSA care [59,62,63].

### 4.3. Circadian Rhythm Disruption and Chrononutrition

Circadian rhythms regulate numerous physiological processes involved in metabolism, including glucose homeostasis, lipid metabolism, hormone secretion, gastrointestinal function, and energy expenditure [78]. OSA disrupts normal circadian organization through recurrent sleep interruptions, altered melatonin secretion, increased nocturnal sympathetic activation, and fluctuations in oxygen availability. These disturbances impair the temporal coordination of endocrine and metabolic pathways, contributing to sleep fragmentation and cardiometabolic dysfunction [19,69,83].

Melatonin plays a central role in circadian regulation and sleep initiation. Its synthesis in the pineal gland is strongly influenced by environmental light exposure, particularly blue-enriched light during the evening, which suppresses melatonin release and delays sleep onset. Nutritional factors also modulate melatonin physiology. Foods such as tart cherries, nuts, seeds, and certain grains contain melatonin or its precursors, while antioxidant-rich diets may enhance endogenous melatonin signaling by reducing oxidative stress [2]. Disrupted melatonin rhythms in OSA may therefore be exacerbated by late-night eating, irregular meal timing, and exposure to artificial light at night, reinforcing the importance of aligning nutritional behaviors with circadian biology [2,19].

The metabolic pathway linking tryptophan to serotonin and subsequently to melatonin represents another key nutritional mechanism influencing sleep regulation. Tryptophan availability depends on dietary intake and macronutrient composition, particularly carbohydrates, which facilitate its transport across the blood–brain barrier [19]. Vitamin B6 acts as an essential cofactor in serotonin and melatonin synthesis, highlighting the relevance of micronutrient adequacy for circadian stability [4]. Impaired tryptophan metabolism may contribute to difficulties in sleep initiation and maintenance, further aggravating sleep fragmentation in OSA [2,19].

Emerging evidence suggests that meal timing may influence both metabolic health and sleep physiology. Late-night eating, prolonged eating windows, irregular meal schedules, and nighttime caloric intake have been associated with impaired glucose tolerance, increased adiposity, systemic inflammation, and poorer sleep quality [40,41,42,43,82,86]. These factors may further aggravate OSA severity by promoting weight gain and worsening metabolic dysfunction [80].

Chrononutrition, the study of interactions between meal timing and circadian biology, has recently emerged as a promising field in sleep medicine. Preliminary studies indicate that aligning food intake with endogenous circadian rhythms may improve insulin sensitivity, reduce inflammatory responses, and enhance metabolic flexibility [86,87]. Time-restricted eating (TRE) has demonstrated favorable effects on body weight, blood pressure, and glycemic control in populations with obesity and metabolic syndrome; however, evidence specifically involving patients with OSA remains scarce, and randomized controlled trials are needed before routine clinical recommendations can be established [40].

TRE may provide metabolic benefits through improved alignment between food intake and circadian rhythms, reduced late-night energy intake, and improved metabolic regulation [40]. However, evidence specifically evaluating TRE in OSA remains limited. Therefore, TRE should currently be considered an investigational behavioral strategy rather than a standardized OSA treatment. When used clinically for weight or metabolic management, the duration of the eating window should be individualized according to nutritional adequacy, comorbidities, medication use, physical activity, sleep schedule, and patient preference [86].

### 4.4. Physical Inactivity, Energy Expenditure, and Weight Gain

Excessive daytime sleepiness, fatigue, and reduced exercise tolerance frequently observed in patients with OSA contribute to decreased levels of physical activity and lower daily energy expenditure [88,89,90]. Reduced participation in structured exercise and habitual movement favors progressive weight gain, increased visceral adiposity, and loss of skeletal muscle mass, all of which further exacerbate respiratory dysfunction during sleep [91].

Physical inactivity also amplifies insulin resistance, chronic inflammation, endothelial dysfunction, and cardiovascular risk [91]. Importantly, combined lifestyle interventions incorporating dietary modification and regular exercise may produce greater improvements in body composition, metabolic health, and OSA severity than either intervention alone [92,93]. Therefore, nutritional management should always be considered within the broader context of comprehensive lifestyle medicine [94].

### 4.5. Effects of OSA Treatment on Dietary Behavior

CPAP effectively reduces apnea–hypopnea events and improves sleep quality; however, its effects on body weight and eating behavior remain controversial. A modest weight gain following CPAP initiation may result from reductions in nocturnal energy expenditure, improved appetite, or decreased sympathetic activation. Conversely, improvements in daytime alertness and cognitive function may facilitate greater adherence to dietary counseling and physical activity programs, particularly when CPAP is combined with structured lifestyle interventions [90].

The introduction of incretin-based therapies has further changed the therapeutic landscape. Beyond promoting substantial weight loss, GLP-1RAs and dual glucose-dependent insulinotropic polypeptide (GIP)/GLP-1 receptor agonists improve appetite regulation, reduce caloric intake, and enhance glycemic control [1,33]. These pharmacological effects may complement nutritional interventions, supporting long-term behavioral changes and potentially improving adherence to dietary recommendations [44,95,96,97].

Taken together, current evidence indicates that nutrition and OSA interact through a dynamic and reciprocal relationship involving neuroendocrine regulation, eating behavior, circadian biology, and energy metabolism. These interactions highlight the need for comprehensive dietary interventions that address not only caloric restriction but also meal quality, food timing, behavioral determinants, and metabolic health. Understanding these mechanisms provides the foundation for evaluating how specific dietary patterns influence OSA outcomes, which is discussed in the following section (Figure 2).

### 4.6. Sleep Hygiene, Environmental Stimuli, and Behavioral Nutrition in OSA

Sleep hygiene and environmental stimuli interact directly with nutritional status, circadian rhythms, and the pathophysiology of OSA [2,19]. Although the manuscript discusses chrononutrition and appetite-regulating hormones, it is essential to integrate behavioral sleep practices that modulate these same biological pathways. Light exposure, meal timing, evening dietary composition, and environmental conditions influence melatonin synthesis, autonomic balance, ventilatory control stability, and upper-airway neuromuscular responsiveness, thereby affecting both sleep architecture and OSA severity [19].

Evening exposure to blue-enriched light suppresses pineal melatonin production and delays circadian phase, increasing sleep latency and reducing REM sleep stability. Because OSA tends to worsen during REM sleep, circadian delays and melatonin suppression may exacerbate respiratory events and arousals [2,19]. Establishing a dim-light environment in the hours preceding bedtime helps maintain endogenous melatonin rhythms and supports the alignment between central and peripheral clocks, which is essential for metabolic homeostasis. From a clinical perspective, these observations support general light-hygiene measures, such as exposure to bright natural light during the daytime and reduction in intense or blue-enriched light exposure during the evening. These measures may support circadian alignment and sleep quality, but they should be regarded as complementary behavioral strategies rather than OSA-specific treatments [19].

Meal timing may influence metabolic and circadian regulation relevant to sleep. Consuming large meals close to bedtime may increase gastrointestinal and metabolic load during the biological night and may be associated with poorer sleep-related outcomes in some populations. Accordingly, a practical approach is to avoid large meals immediately before bedtime and to favor a regular, daytime-oriented eating schedule when compatible with individual needs [40]. However, an optimal interval between the last meal and bedtime has not been established specifically for OSA, and controlled studies are needed before a universal timing recommendation can be made. Alcohol intake in the evening further compounds these effects. Alcohol reduces upper-airway dilator muscle tone, increases pharyngeal collapsibility, and destabilizes ventilatory control, leading to more frequent apneas and deeper oxygen desaturations. It also fragments REM sleep, reinforcing the cycle of sleep disruption and metabolic impairment. Similarly, high-fat and high-sugar foods consumed at night impair leptin signaling, increase nocturnal reflux, and elevate sympathetic activity, all of which contribute to sleep fragmentation and exacerbate OSA [40,58,87].

Maintaining a sleep–wake schedule strengthens circadian alignment and stabilizes appetite-regulating hormones such as ghrelin, leptin, GLP-1, and PYY [1]. Regularity in sleep timing improves adherence to structured feeding windows and supports metabolic homeostasis, reducing the likelihood of evening overeating and weight gain, key contributors to OSA progression. Environmental optimization, including a dark, quiet, and cool bedroom, reduces arousal threshold variability and supports ventilatory stability. These environmental cues also reinforce behavioral nutrition strategies by facilitating predictable routines around meal timing and sleep preparation [19]. Collectively, these behavioral and environmental practices interact synergistically with nutritional interventions. They reduce nighttime arousals, improve melatonin synthesis, stabilize autonomic balance, enhance insulin sensitivity, and support weight management [2,19]. When combined with dietary patterns, CPAP therapy, physical activity, and incretin-based treatments, sleep hygiene becomes an essential component of a comprehensive, multidisciplinary approach to OSA management.

## 5. Dietary Patterns and Clinical Evidence in Obstructive Sleep Apnea

Dietary modification has become a cornerstone of lifestyle management in obesity-related disorders, including OSA [56,57,58,59,60,61]. While weight reduction remains the primary therapeutic goal, growing evidence suggests that dietary quality, food composition, and eating patterns influence OSA severity through mechanisms extending beyond body weight alone [62,63,64,95]. Different dietary patterns exert distinct effects on systemic inflammation, oxidative stress, endothelial function, insulin sensitivity, gut microbiota, and cardiometabolic health, all of which play important roles in OSA pathophysiology [38,41,71,96]. Nevertheless, evidence directly evaluating the impact of specific dietary patterns on OSA remains relatively limited compared with other cardiometabolic diseases, and most available studies are characterized by small sample sizes, heterogeneous interventions, and relatively short follow-up periods [98].

### 5.1. Mediterranean Diet

Among all dietary approaches, the Mediterranean diet has received the greatest attention because of its well-established cardiovascular and metabolic benefits [61,89]. Characterized by a high intake of vegetables, fruits, legumes, whole grains, nuts, olive oil, and fish, moderate consumption of dairy products, and limited intake of red and processed meats, the Mediterranean diet is rich in monounsaturated fatty acids, dietary fiber, antioxidants, and polyphenols that collectively reduce oxidative stress and chronic inflammation [2,99].

Several observational studies have demonstrated an inverse association between adherence to the Mediterranean diet and OSA severity, independent of body mass index [2,99]. Individuals with higher Mediterranean Diet Scores generally exhibit lower apnea–hypopnea index (AHI), reduced daytime sleepiness, improved insulin sensitivity, and better cardiovascular risk profiles [61]. Although these associations do not establish causality, they suggest that dietary quality may influence disease severity beyond its effects on body weight [99].

Randomized controlled trials have provided additional support for these findings. The landmark study by Papandreou and colleagues demonstrated that a calorie-restricted Mediterranean diet combined with CPAP resulted in greater reductions in AHI during REM sleep, improved waist circumference, and superior adherence to lifestyle modification compared with a prudent diet plus CPAP [90,100]. These improvements were accompanied by significant reductions in visceral adiposity, supporting the hypothesis that body fat distribution may be more relevant than overall weight loss in modifying upper airway function [101].

The biological mechanisms underlying these benefits are likely multifactorial. Olive oil-derived monounsaturated fatty acids improve endothelial function through enhanced nitric oxide bioavailability, whereas polyphenols attenuate reactive oxygen species production and inhibit NF-κB-mediated inflammatory signaling [102]. Furthermore, increased consumption of dietary fiber promotes the production of short-chain fatty acids by the gut microbiota, contributing to improved insulin sensitivity and reduced systemic inflammation [38,41,71,96,103].

Despite these promising findings, most intervention studies evaluating the Mediterranean diet in OSA remain relatively small and frequently combine dietary modification with caloric restriction, physical activity, or CPAP, making it difficult to isolate the independent effects of dietary composition [90,104].

### 5.2. Dietary Approaches to Stop Hypertension (DASH) Diet

The Dietary Approaches to Stop Hypertension (DASH) diet has been extensively investigated as a non-pharmacological strategy for reducing blood pressure and improving cardiovascular health [50]. Similar to the Mediterranean diet, the DASH pattern emphasizes fruits, vegetables, whole grains, legumes, low-fat dairy products, and lean protein sources while limiting sodium, refined carbohydrates, sugar-sweetened beverages, and saturated fat [35,42,105,106].

Although relatively few studies have specifically evaluated the DASH diet in OSA populations, its physiological effects directly target several mechanisms implicated in disease progression. Improved endothelial function, lower systemic inflammation, enhanced insulin sensitivity, and reductions in arterial stiffness may collectively attenuate the cardiovascular burden associated with OSA. Furthermore, the high potassium, magnesium, and calcium content of the DASH diet contributes to improved blood pressure regulation, an important consideration given the high prevalence of resistant hypertension among patients with OSA [35,42,50,107].

### 5.3. Plant-Based Dietary Patterns

Plant-based dietary patterns, including vegetarian and predominantly plant-based diets, have gained increasing attention because of their anti-inflammatory and cardiometabolic properties [5]. These dietary approaches typically provide higher amounts of dietary fiber, unsaturated fatty acids, polyphenols, vitamins, and minerals while reducing intake of saturated fats and processed foods [54,56].

Higher consumption of plant-derived foods has been associated with lower body weight, improved insulin sensitivity, reduced visceral adiposity, and decreased circulating inflammatory biomarkers, all of which are mechanistically relevant to OSA [5]. In addition, plant-rich diets favor greater microbial diversity and increased production of short-chain fatty acids, promoting intestinal barrier integrity and immunometabolic homeostasis [54,56].

Nevertheless, direct evidence linking plant-based diets with improvements in OSA severity remains limited. Most available studies have examined sleep quality rather than objective respiratory outcomes, highlighting the need for prospective clinical trials evaluating polysomnographic endpoints.

### 5.4. Low-Carbohydrate and Ketogenic Diets

Low-carbohydrate diets have become increasingly popular for obesity treatment because of their effectiveness in promoting short-term weight loss and improving glycemic control [86]. Ketogenic diets, characterized by severe carbohydrate restriction and increased fat intake, induce nutritional ketosis, which enhances lipolysis and reduces circulating insulin concentrations [87]. Rapid weight loss induced by these dietary approaches may reduce upper airway fat deposition and improve respiratory mechanics, potentially decreasing OSA severity [108,109,110,111,112,113]. Furthermore, improved insulin sensitivity and reductions in systemic inflammation may contribute to additional metabolic benefits [70,101].

However, concerns remain regarding the long-term sustainability and cardiovascular safety of ketogenic diets, particularly when saturated fat intake is excessive [114]. Current evidence does not support ketogenic diets as superior to other calorie-restricted dietary patterns for long-term OSA management. Rather, overall dietary adherence appears to be a more important determinant of sustained weight loss and clinical improvement than macronutrient distribution alone [104,115].

### 5.5. Chrononutrition and Time-Restricted Eating

Meal timing has recently emerged as an important determinant of metabolic health. TRE, which limits daily food intake to a defined eating window without necessarily prescribing caloric restriction, has demonstrated favorable effects on body weight, insulin sensitivity, blood pressure, and inflammatory biomarkers in individuals with obesity and metabolic syndrome [40].

The potential relevance of TRE in OSA extends beyond weight reduction. Synchronizing food intake with endogenous circadian rhythms may improve metabolic flexibility, reduce nocturnal hyperglycemia, and optimize hormonal regulation of appetite [17]. Experimental evidence also suggests that avoiding late-night meals may reduce nocturnal gastroesophageal reflux and improve sleep quality, although studies specifically involving OSA remain limited [40,41,42]. Given the intimate relationship between circadian disruption and OSA pathophysiology, chrononutrition represents a promising area for future investigation. Nevertheless, randomized controlled trials are needed before specific recommendations regarding meal timing can be incorporated into routine OSA management [40].

### 5.6. Western Dietary Patterns and Ultra-Processed Foods

In contrast to healthy dietary patterns, Western diets characterized by high consumption of ultra-processed foods, refined grains, processed meats, sugar-sweetened beverages, and saturated fats promote systemic inflammation, oxidative stress, insulin resistance, and obesity. These dietary characteristics closely mirror the biological mechanisms underlying OSA progression [2,62].

Ultra-processed foods are generally energy-dense, highly palatable, and poor in fiber and micronutrients, favoring excessive caloric intake and visceral adiposity. Moreover, food additives, emulsifiers, and artificial sweeteners may alter gut microbiota composition and increase intestinal permeability, further amplifying systemic inflammatory responses [21,109,110,111,112,113].

Emerging epidemiological evidence suggests that greater consumption of ultra-processed foods is independently associated with poorer sleep quality, shorter sleep duration, and increased risk of sleep-disordered breathing [22]. Although causality has not yet been established, these findings reinforce current recommendations emphasizing minimally processed dietary patterns in patients with OSA [114,115,116,117,118,119].

### 5.7. Comparative Perspective

Collectively, available evidence indicates that no single dietary pattern can currently be considered the definitive nutritional therapy for OSA. Instead, dietary approaches characterized by high consumption of minimally processed plant foods, healthy unsaturated fats, whole grains, and dietary fiber may demonstrate the greatest potential to improve the metabolic and inflammatory pathways involved in disease pathophysiology. Among the available options, the Mediterranean diet currently presents the strongest body of evidence, followed by the DASH diet, whereas evidence supporting ketogenic diets, TRE, and plant-based interventions remains promising but considerably less robust [2,5,17,40,42,54,56,86,87,104,108,109,110,111,112,113,114,115].

Importantly, future dietary recommendations should move beyond universal prescriptions toward individualized nutritional strategies that consider obesity phenotype, metabolic profile, cardiovascular risk, gut microbiota composition, and patient preferences. This personalized approach aligns with the emerging paradigm of precision nutrition and may ultimately optimize long-term adherence and therapeutic effectiveness in OSA management.

While dietary patterns provide an overall framework for nutritional management, individual nutrients and bioactive compounds may also directly influence the biological mechanisms underlying OSA. Understanding how specific micronutrients, fatty acids, antioxidants, polyphenols, and other dietary components modulate inflammation, oxidative stress, endothelial function, and neuromuscular regulation may further support the development of targeted nutritional interventions. These aspects are discussed in the following section (Table 2).

## 6. Micronutrients and Bioactive Compounds: Emerging Targets for Nutritional Management of OSA

Although overall dietary patterns exert broad effects on metabolic health, increasing evidence suggests that individual micronutrients and bioactive compounds may directly influence several biological pathways implicated in obstructive sleep apnea (OSA), including oxidative stress, chronic inflammation, endothelial dysfunction, mitochondrial homeostasis, immune regulation, and neuromuscular function [4,23,24,116]. Nevertheless, unlike established dietary patterns such as the Mediterranean diet, evidence supporting isolated nutrient supplementation in OSA remains inconsistent. Current data generally favor achieving adequate nutrient intake through healthy dietary patterns rather than routine supplementation, except in individuals with documented deficiencies or specific clinical indications [25,117].

### 6.1. Vitamin D

Vitamin D is among the most extensively investigated micronutrients in sleep medicine because of its pleiotropic effects on immune regulation, skeletal muscle function, inflammation, and cardiovascular health [111]. Beyond its classical role in calcium homeostasis, vitamin D receptors are widely expressed in skeletal muscle, vascular endothelium, adipose tissue, and several brain regions involved in sleep regulation and respiratory control [2,4,27,117].

Lower serum 25-hydroxyvitamin D concentrations in patients with OSA compared with healthy controls, with deficiency appearing more common among individuals with severe disease. Furthermore, lower vitamin D levels have been associated with higher AHI, poorer sleep quality, excessive daytime sleepiness, and increased circulating inflammatory biomarkers. However, obesity represents an important confounding factor, as vitamin D is sequestered within adipose tissue, reducing its bioavailability independent of OSA severity [111].

Several mechanisms may explain the relationship between vitamin D and OSA. Vitamin D modulates innate and adaptive immune responses by suppressing nuclear factor-kappa B (NF-κB) activation and reducing the production of pro-inflammatory cytokines, including interleukin-6 (IL-6) and tumor necrosis factor-alpha (TNF-α) [2,4]. Additionally, vitamin D supports skeletal muscle strength and neuromuscular coordination, raising the hypothesis that deficiency may impair upper airway dilator muscle function and increase pharyngeal collapsibility during sleep [111].

Despite these biological plausibility arguments, randomized controlled trials evaluating vitamin D supplementation have produced conflicting results. While some studies have demonstrated modest improvements in inflammatory biomarkers and subjective sleep quality, possible reductions in AHI have not been observed [111]. Current evidence therefore does not support routine vitamin D supplementation as a treatment for OSA, although correction of deficiency remains appropriate according to established endocrine guidelines [2,4].

### 6.2. Omega-3 Polyunsaturated Fatty Acids

Long-chain omega-3 polyunsaturated fatty acids (PUFAs), particularly eicosapentaenoic acid (EPA) and docosahexaenoic acid (DHA), possess well-established anti-inflammatory, antioxidant, and cardioprotective properties [99,112]. These fatty acids regulate membrane fluidity, improve endothelial nitric oxide production, inhibit platelet aggregation, and promote the synthesis of specialized pro-resolving mediators, including resolvins, protectins, and maresins, which actively terminate inflammatory responses [2].

Studies suggest that omega-3 PUFAs may attenuate intermittent hypoxia-induced oxidative stress and vascular dysfunction through reduced reactive oxygen species generation and improved mitochondrial function [2]. Additionally, omega-3 fatty acids appear to improve autonomic balance by enhancing parasympathetic activity and reducing sympathetic nervous system activation, mechanisms potentially relevant to cardiovascular complications associated with OSA [99,112].

Clinical evidence, however, remains limited. Small observational studies have reported inverse associations between circulating DHA concentrations and OSA severity, whereas intervention trials specifically evaluating omega-3 supplementation in OSA populations are scarce [2,105]. Given their established cardiovascular benefits, increasing dietary intake of oily fish, walnuts, flaxseed, and other omega-3-rich foods appears reasonable as part of an overall cardioprotective dietary strategy, although direct effects on respiratory outcomes require further investigation [109,110].

### 6.3. Dietary Fiber and Short-Chain Fatty Acids

Dietary fiber represents one of the most important nutritional determinants of metabolic health because of its effects on glycemic control, satiety, body weight regulation, and gut microbiota composition [47]. Fermentation of soluble fiber by intestinal microorganisms produces short-chain fatty acids (SCFAs), including acetate, propionate, and butyrate, which exert profound effects on host metabolism and immune function [2,40].

SCFAs improve intestinal barrier integrity, reduce endotoxemia, suppress inflammatory signaling, and enhance insulin sensitivity through activation of G-protein-coupled receptors and inhibition of histone deacetylases [111,112,113,114,115,116,117,118]. These mechanisms are particularly relevant in OSA, where chronic intermittent hypoxia has been associated with gut dysbiosis, increased intestinal permeability, and systemic inflammation [2,42].

Higher dietary fiber intake has been associated with lower body weight, improved glycemic control, reduced circulating CRP, and lower cardiovascular risk [43]. Although direct studies in OSA remain scarce, dietary fiber likely contributes to disease improvement through both weight-dependent and weight-independent mechanisms [47].

### 6.4. Polyphenols and Other Dietary Antioxidants

Polyphenols comprise a diverse group of bioactive compounds naturally present in fruits, vegetables, tea, cocoa, coffee, extra-virgin olive oil, herbs, and spices [2]. These compounds exert antioxidant, anti-inflammatory, vasoprotective, and metabolic effects through multiple molecular pathways [47].

Among their best-characterized mechanisms is activation of nuclear factor erythroid 2-related factor 2 (Nrf2), a transcription factor that regulates endogenous antioxidant defense systems [45]. Polyphenols also inhibit NF-κB activation, improve endothelial nitric oxide bioavailability, reduce mitochondrial oxidative stress, and modulate gut microbiota composition [46].

Experimental models of intermittent hypoxia have demonstrated that several polyphenols, including resveratrol, quercetin, epigallocatechin gallate (EGCG), and curcumin, attenuate oxidative stress, vascular remodeling, sympathetic activation, and inflammatory signaling [73,74]. However, clinical evidence in patients with OSA remains extremely limited, and most available studies involve animal models or populations with obesity rather than confirmed sleep-disordered breathing. Accordingly, current evidence supports increasing consumption of naturally polyphenol-rich foods within healthy dietary patterns rather than recommending isolated nutraceutical supplementation [2].

### 6.5. Magnesium

Magnesium is an essential intracellular cation involved in more than 300 enzymatic reactions related to energy metabolism, neuromuscular transmission, glucose regulation, and vascular function [4]. Inadequate magnesium intake has been associated with systemic inflammation, insulin resistance, endothelial dysfunction, hypertension, and impaired sleep quality [2].

Studies suggest that lower dietary magnesium intake is associated with shorter sleep duration and poorer sleep quality in the general population [4]. Although evidence specifically involving OSA remains limited, magnesium deficiency may contribute indirectly to disease progression by worsening metabolic dysfunction and cardiovascular risk [2]. Current data do not support magnesium supplementation specifically for OSA treatment. Nevertheless, adequate intake through magnesium-rich foods, including legumes, nuts, seeds, whole grains, and leafy green vegetables, should be encouraged as part of a cardiometabolic dietary pattern.

### 6.6. Vitamin B6 and Sleep Neurotransmitter Synthesis

Vitamin B6 (pyridoxine) is a critical cofactor in the conversion of tryptophan to serotonin and subsequently to melatonin. Deficiency may impair sleep initiation, reduce sleep efficiency, and alter REM sleep regulation. Because OSA is associated with sleep fragmentation and dysregulated neurotransmitter pathways, ensuring adequate vitamin B6 intake through foods such as fish, poultry, bananas, and whole grains may support sleep quality and circadian alignment. Although direct evidence in OSA is limited, the mechanistic role of vitamin B6 in melatonin synthesis provides a strong biological rationale for its inclusion in nutritional recommendations [2,19,114].

### 6.7. Iron and Upper Airway Muscle Tone

Iron plays a central role in oxygen transport, mitochondrial function, and neuromuscular activity. Deficiency may impair upper airway muscle tone and contribute to sleep disturbances, as observed in conditions such as restless legs syndrome. In OSA, iron deficiency may exacerbate fatigue, sleep fragmentation, and impaired ventilatory control. Dietary sources such as lean meats, legumes, and fortified grains, combined with adequate vitamin C intake to enhance absorption, may help maintain iron homeostasis and support respiratory muscle function [2,19,107].

### 6.8. N-Acetylcysteine: Antioxidant and Mucolytic Effects Relevant to OSA

N-acetylcysteine (NAC) is a precursor to glutathione and exhibits potent antioxidant and mucolytic properties. NAC may reduce oxidative stress induced by intermittent hypoxia, improve endothelial function, and enhance upper airway muscle stability [85]. Preliminary evidence suggests potential benefits for sleep quality and respiratory function, although OSA-specific trials remain scarce. Given the limited number of OSA-specific clinical studies, NAC should currently be regarded as an investigational bioactive compound rather than an established nutritional therapy for OSA [2,19,85].

### 6.9. Emerging Nutritional Compounds

Growing interest has focused on several additional bioactive compounds that may influence OSA-related pathophysiology, although evidence remains preliminary [2]. Melatonin, traditionally recognized as a regulator of circadian rhythms, also exhibits antioxidant and anti-inflammatory properties that may attenuate intermittent hypoxia-induced oxidative damage [2,51,52]. Likewise, dietary nitrates derived from beetroot and green leafy vegetables improve nitric oxide bioavailability and endothelial function, potentially counteracting vascular dysfunction associated with OSA [52]. Other compounds, including coenzyme Q10, probiotics, prebiotics, carotenoids, and various phytochemicals, have demonstrated promising results in experimental models but currently lack sufficient clinical evidence for routine recommendation [4,113].

Although these compounds have biological plausibility and have been investigated in experimental or non-OSA populations, current evidence is insufficient to support their routine use as OSA-specific therapies. Their potential clinical relevance should therefore be considered investigational and requires validation in well-designed human studies with objective sleep and respiratory outcomes.

### 6.10. Clinical Perspective

Despite increasing interest in individual nutrients, current evidence indicates that the benefits of nutrition in OSA are primarily attributable to the synergistic effects of whole dietary patterns rather than isolated micronutrients or supplements. Nutrients interact within complex food matrices, and their biological effects are influenced by dietary composition, gut microbiota, genetic background, metabolic phenotype, and overall lifestyle.

Consequently, nutritional management should prioritize food-based interventions emphasizing minimally processed plant foods, healthy unsaturated fats, dietary fiber, and naturally occurring bioactive compounds instead of relying on high-dose supplementation. Personalized supplementation may be appropriate in selected patients with documented deficiencies, but routine use of isolated nutraceuticals cannot currently be recommended based on available evidence.

Healthy dietary patterns may influence gut microbial composition and function, providing a plausible pathway linking dietary quality with metabolic and inflammatory processes relevant to OSA. However, the extent to which microbiota changes mediate clinical respiratory outcomes in humans remains uncertain. Therefore, microbiota modulation should be considered a potential mechanism and research target rather than an established component of OSA-specific nutritional therapy. The following section examines the growing evidence supporting the role of the gut–lung axis in OSA and its potential as a therapeutic target through nutritional modulation (Table 3).

## 7. Gut Microbiota: A Novel Link Between Nutrition and Obstructive Sleep Apnea

The human gut microbiota has emerged as a critical regulator of metabolic homeostasis, immune function, cardiovascular health, and sleep physiology [38]. Comprising trillions of microorganisms, the intestinal microbiome actively participates in nutrient metabolism, maintenance of epithelial barrier integrity, modulation of inflammatory responses, and production of bioactive metabolites that influence distant organs through complex endocrine, neural, and immune pathways [41]. Increasing evidence suggests that alterations in gut microbial composition, collectively referred to as gut dysbiosis, contribute to the pathogenesis of obesity, insulin resistance, hypertension, and cardiovascular disease, all of which frequently coexist with OSA [71]. Consequently, the gut microbiota has emerged as a potential mechanistic bridge linking dietary habits with OSA development and its systemic complications.

### 7.1. Intermittent Hypoxia Induces Gut Dysbiosis

Intermittent hypoxia, a defining characteristic of OSA, extends its physiological effects beyond the respiratory and cardiovascular systems to profoundly influence intestinal homeostasis. Experimental animal models have demonstrated that recurrent hypoxia–reoxygenation cycles alter gut microbial diversity, reduce beneficial bacterial populations, increase the abundance of pro-inflammatory microorganisms, and disrupt intestinal barrier integrity [58].

One of the earliest consequences of intermittent hypoxia is a reduction in microbial diversity, a hallmark of gut dysbiosis associated with numerous chronic diseases [59]. These microbial alterations are accompanied by decreased production of beneficial metabolites, particularly SCFAs, and increased proliferation of Gram-negative bacteria capable of producing lipopolysaccharide (LPS), a potent endotoxin that promotes systemic inflammation [71]. Importantly, these alterations appear to occur independently of obesity, indicating that intermittent hypoxia itself directly influences microbial ecology. Human studies have similarly reported significant differences in gut microbial composition between patients with OSA and healthy controls, although considerable interindividual variability remains and no universal microbial signature has yet been identified [37,41].

### 7.2. Increased Intestinal Permeability and Metabolic Endotoxemia

Gut dysbiosis contributes to disruption of the intestinal epithelial barrier, allowing bacterial products such as LPS to translocate into the systemic circulation, a phenomenon commonly referred to as metabolic endotoxemia. Increased circulating LPS activates Toll-like receptor 4 (TLR4) signaling on immune cells, initiating downstream activation of nuclear factor-kappa B (NF-κB) and stimulating production of pro-inflammatory cytokines including tumor necrosis factor-alpha (TNF-α), interleukin-6 (IL-6), and interleukin-1β (IL-1β) [41].

This chronic inflammatory state contributes to endothelial dysfunction, insulin resistance, adipose tissue inflammation, vascular remodeling, and hypertension, thereby amplifying the cardiometabolic burden associated with OSA. Furthermore, systemic inflammation may impair upper airway neuromuscular function and increase tissue edema, potentially worsening pharyngeal collapsibility and disease severity. The interaction between intermittent hypoxia, gut barrier dysfunction, and systemic inflammation creates a self-reinforcing pathogenic cycle in which OSA promotes dysbiosis, while dysbiosis exacerbates metabolic dysfunction and cardiovascular complications [71].

### 7.3. Short-Chain Fatty Acids as Key Mediators

Among the numerous metabolites produced by intestinal microorganisms, SCFAs, primarily acetate, propionate, and butyrate, have received considerable attention because of their broad metabolic and immunological effects [38]. SCFAs serve as the principal energy source for colonocytes, strengthen epithelial tight junctions, regulate intestinal permeability, and suppress inflammatory signaling through activation of G-protein-coupled receptors (GPR41 and GPR43) and inhibition of histone deacetylases [41].

Beyond the gastrointestinal tract, SCFAs improve insulin sensitivity, regulate lipid metabolism, enhance secretion of GLP-1 and peptide YY (PYY), reduce oxidative stress, and modulate autonomic nervous system activity. Experimental studies further suggest that SCFAs influence central nervous system function through the gut–brain axis, potentially affecting sleep regulation and circadian physiology [41]. Because healthy dietary patterns rich in dietary fiber promote SCFA production, nutritional interventions aimed at restoring microbial diversity may provide multiple benefits extending beyond body weight reduction. Nevertheless, direct evidence linking SCFA modulation to improvements in OSA severity remains limited, representing an important area for future investigation [71].

### 7.4. Diet, Microbiota, and OSA

Diet is widely recognized as one of the strongest determinants of gut microbial composition. Diets rich in fruits, vegetables, legumes, whole grains, and other fiber-containing foods promote greater microbial diversity and increase the abundance of beneficial taxa such as *Bifidobacterium*, *Lactobacillus*, *Faecalibacterium prausnitzii*, and *Akkermansia muciniphila* [69]. These microorganisms contribute to enhanced SCFA production, improved intestinal barrier function, and attenuation of systemic inflammation [2].

Conversely, Western dietary patterns characterized by excessive intake of saturated fats, refined sugars, and ultra-processed foods reduce microbial diversity and favor expansion of pro-inflammatory bacterial species [41]. High-fat diets also increase intestinal permeability and circulating endotoxin concentrations, thereby amplifying inflammatory pathways already activated by intermittent hypoxia [56].

The Mediterranean diet appears particularly effective in promoting a favorable microbial profile because of its high content of dietary fiber, polyphenols, and unsaturated fatty acids [2,71]. Polyphenols are metabolized by intestinal bacteria into biologically active compounds that further modulate microbial composition, creating a reciprocal relationship between diet and microbiota. This interaction may partially explain why Mediterranean dietary patterns reduce inflammatory biomarkers even when weight loss is modest [2].

### 7.5. The Gut–Lung Axis in OSA

The concept of the gut–lung axis has emerged as an important paradigm in respiratory medicine. Bidirectional communication between intestinal microorganisms and pulmonary tissues occurs through microbial metabolites, circulating cytokines, immune cell trafficking, and neuroendocrine signaling. Although initially investigated in asthma and chronic obstructive pulmonary disease, the gut–lung axis is increasingly recognized as relevant to OSA. Gut-derived inflammatory mediators may influence pulmonary vascular remodeling, systemic oxidative stress, and immune activation, whereas intermittent hypoxia alters intestinal oxygen gradients and microbial ecology [71]. These reciprocal interactions suggest that modulation of gut microbiota may eventually become an adjunctive therapeutic strategy in sleep medicine.

### 7.6. Probiotics, Prebiotics, and Synbiotics

Interest in microbiota-targeted therapies has increased substantially over the past decade. Probiotics, prebiotics, and synbiotics have demonstrated beneficial effects in obesity, metabolic syndrome, and type 2 diabetes by improving microbial diversity, reducing systemic inflammation, and enhancing insulin sensitivity [2]. However, evidence specifically involving OSA remains scarce. Only a limited number of small clinical studies have evaluated probiotic supplementation in patients with sleep disorders, and robust improvements in apnea severity have not yet been demonstrated [41]. Therefore, current evidence does not support routine probiotic supplementation as a treatment for OSA. Instead, dietary strategies naturally promoting microbial diversity, including increased intake of dietary fiber, legumes, fruits, vegetables, fermented foods, and polyphenol-rich foods, currently represent the most evidence-based approach for modulating gut microbiota in clinical practice [2,113].

### 7.7. Future Perspectives: Toward Microbiome-Based Precision Nutrition

Rapid advances in metagenomics, metabolomics, and systems biology have transformed the understanding of the gut microbiome from a descriptive field into a promising component of precision medicine. Individual differences in microbial composition influence responses to dietary interventions, glucose metabolism, body weight regulation, inflammatory status, and drug metabolism, suggesting that future nutritional recommendations may increasingly incorporate microbiome profiling.

In OSA, microbiome-based precision nutrition represents an emerging concept with considerable potential. Integrating microbial signatures with clinical phenotypes, inflammatory biomarkers, metabolomic profiles, and cardiometabolic characteristics may facilitate individualized dietary interventions that maximize therapeutic response while minimizing unnecessary dietary restrictions. Although these approaches remain largely investigational, ongoing advances in artificial intelligence, machine learning, and multi-omics technologies are expected to accelerate the development of personalized nutrition strategies in sleep medicine over the coming decade.

Collectively, current evidence identifies the gut microbiota as a central mediator linking dietary habits with inflammation, metabolic dysfunction, endothelial injury, and cardiovascular risk in OSA. While microbiome-targeted therapies remain in their infancy, dietary modulation of microbial ecology represents a biologically plausible and clinically accessible strategy for improving systemic health. Importantly, these advances also reinforce the broader concept of precision nutrition, in which nutritional recommendations are tailored according to individual biological characteristics rather than generalized dietary prescriptions. This paradigm is further strengthened by the recent emergence of incretin-based therapies, which are reshaping the management of obesity and OSA and are discussed in the following section (Figure 3).

## 8. Precision Nutrition and Incretin-Based Therapies: Toward Personalized Management of Obstructive Sleep Apnea

The recognition that OSA is a heterogeneous disorder has fundamentally changed the therapeutic paradigm from a “one-size-fits-all” approach toward individualized patient management [77]. Although CPAP remains the standard treatment for moderate-to-severe OSA, considerable interindividual variability exists regarding disease severity, symptom burden, cardiometabolic risk, and treatment responsiveness [100,119]. These observations have stimulated growing interest in precision medicine, an approach that seeks to tailor therapeutic interventions according to individual biological, physiological, genetic, and environmental characteristics [79].

Within this evolving framework, precision nutrition has emerged as a complementary strategy capable of integrating nutritional interventions with metabolic phenotypes, inflammatory status, gut microbiota composition, and behavioral characteristics. Rather than prescribing identical dietary recommendations for all patients with OSA, precision nutrition recognizes that individual responses to dietary interventions vary substantially according to complex interactions among genetics, metabolism, microbiome composition, circadian biology, and lifestyle [80].

### 8.1. Precision Nutrition: Concept and Clinical Relevance

Precision nutrition has been defined as the development of personalized dietary recommendations based on individual biological characteristics to optimize health outcomes and prevent disease [81]. Unlike conventional nutritional guidelines that primarily target population averages, precision nutrition incorporates multidimensional information, including genetic variants, metabolomic profiles, microbiome composition, body composition, inflammatory biomarkers, dietary behavior, and environmental exposures [82]. This concept appears particularly relevant in OSA because the disorder encompasses multiple clinical phenotypes and physiological endotypes. Two patients with similar AHI values may exhibit markedly different degrees of obesity, insulin resistance, systemic inflammation, visceral adiposity, cardiovascular risk, and treatment response [83]. Consequently, nutritional strategies should ideally be individualized according to each patient’s predominant biological characteristics rather than relying solely on disease severity. For example, patients with severe visceral obesity and insulin resistance may derive greater benefit from aggressive weight-loss interventions combined with incretin-based therapies, whereas lean individuals with prominent craniofacial abnormalities and minimal metabolic dysfunction may require different therapeutic priorities. Similarly, patients with marked systemic inflammation or gut dysbiosis may benefit from dietary approaches emphasizing anti-inflammatory foods, increased dietary fiber, and microbiota modulation [84].

Within this broader framework, the terms personalized nutrition, individualized nutrition, and nutrigenomics are related but not interchangeable. Personalized nutrition is a broader concept commonly used to describe dietary recommendations adapted to an individual’s characteristics and needs, whereas individualized nutrition emphasizes the practical adaptation of dietary interventions to the patient’s specific nutritional requirements, preferences, behaviors, and clinical context. Nutrigenomics, in turn, represents a specific scientific field that investigates the interactions between diet or nutrients and the genome, including how genetic variation may influence individual responses to dietary exposures. These concepts are complementary rather than synonymous, with precision nutrition providing an overarching framework that may incorporate personalized and individualized approaches, as well as nutrigenomic and other biological information, to support more targeted nutritional strategies.

### 8.2. OSA Phenotypes and Nutritional Implications

OSA is increasingly understood as a syndrome encompassing multiple phenotypes with distinct clinical and pathophysiological characteristics [85]. Traditional classification based exclusively on AHI inadequately reflects disease complexity and fails to predict therapeutic responsiveness or long-term cardiovascular outcomes. Several clinically relevant phenotypes have been proposed, including obesity-related OSA, positional OSA, REM-predominant OSA, insomnia-associated OSA, minimally symptomatic OSA, and OSA accompanied by severe cardiometabolic dysfunction [86]. Each phenotype may require different nutritional priorities. 

Patients with obesity-related OSA often exhibit marked insulin resistance, visceral adiposity, metabolic syndrome, and chronic inflammation, making structured weight-loss interventions central to management [87]. In contrast, individuals with normal body weight but severe anatomical susceptibility may derive comparatively smaller benefits from caloric restriction alone, emphasizing the importance of comprehensive phenotyping before initiating nutritional therapy. Increasing attention has also been directed toward metabolic phenotypes characterized by varying degrees of hepatic steatosis, sarcopenic obesity, dyslipidemia, and systemic inflammation [74]. These metabolic characteristics may influence dietary responsiveness independently of BMI, supporting a shift toward body composition analysis and metabolic profiling during nutritional assessment [88,89,90,91].

### 8.3. Incretin-Based Therapies: A New Era in OSA Management

The introduction of incretin-based pharmacotherapy represents one of the most important recent advances in obesity medicine and has profound implications for OSA management [92,93,94]. GLP-1RAs, including liraglutide and semaglutide, together with dual GIP/GLP-1 receptor agonists such as tirzepatide, produce unprecedented reductions in body weight while simultaneously improving glycemic control, systemic inflammation, blood pressure, and cardiovascular risk. These agents reduce appetite through central hypothalamic pathways, delay gastric emptying, improve insulin sensitivity, and promote substantial reductions in visceral adiposity. Importantly, reductions in upper airway fat deposition may decrease pharyngeal collapsibility, providing a plausible mechanism through which weight loss translates into improvements in OSA severity [95].

The phase III SURMOUNT-OSA trials recently demonstrated that tirzepatide significantly reduced AHI, body weight, hypoxic burden, and patient-reported symptoms in adults with obesity and moderate-to-severe OSA, representing the first pharmacological therapy specifically approved for OSA associated with obesity [95,97]. These findings mark a paradigm shift in sleep medicine by demonstrating that pharmacological treatment targeting obesity can directly improve respiratory outcomes rather than solely reducing cardiometabolic risk. Nevertheless, weight loss alone probably does not fully explain the observed improvements. Incretin-based therapies also reduce systemic inflammation, improve endothelial function, decrease ectopic fat accumulation, enhance insulin sensitivity, and modulate autonomic nervous system activity, suggesting that multiple biological pathways contribute to therapeutic efficacy [96].

### 8.4. Integrating Nutrition with Incretin Therapy

Despite the remarkable efficacy of incretin-based medications, pharmacotherapy should not be considered a substitute for nutritional intervention. Instead, dietary management remains fundamental for maximizing therapeutic response, preserving lean body mass during rapid weight loss, optimizing micronutrient intake, and supporting long-term weight maintenance [96]. Patients receiving GLP-1RAs frequently experience reduced appetite and substantially lower caloric intake. Without appropriate nutritional counseling, these changes may inadvertently reduce protein, vitamin, mineral, and fiber intake, increasing the risk of inadequate nutrient consumption, loss of skeletal muscle mass, and gastrointestinal adverse effects [95]. Consequently, nutritional interventions accompanying incretin therapy should emphasize adequate dietary protein intake to preserve muscle mass, sufficient fiber consumption to support gastrointestinal function and gut microbiota health, and high-quality dietary patterns rich in minimally processed foods. Particular attention should also be paid to hydration, micronutrient adequacy, and resistance exercise to minimize treatment-related sarcopenia [96]. This integrated approach reinforces the concept that pharmacological and nutritional therapies should be viewed as complementary rather than competing strategies [97].

### 8.5. Biomarkers for Personalized Nutritional Care

One of the major goals of precision nutrition is identifying biomarkers capable of predicting individual responses to dietary interventions [98]. Several candidate biomarkers may eventually contribute to personalized nutritional management in OSA. Anthropometric indicators, including waist circumference, neck circumference, visceral adipose tissue, and body composition assessed by dual-energy X-ray absorptiometry (DXA) or bioelectrical impedance analysis, provide more informative measures than BMI alone [99]. Likewise, inflammatory biomarkers such as CRP, IL-6, TNF-α, and adipokines may identify patients who would benefit from anti-inflammatory dietary interventions. Metabolic biomarkers, including fasting insulin, HOMA-IR, glycated hemoglobin, triglyceride-to-HDL cholesterol ratio, liver fat quantification, and continuous glucose monitoring, may further assist in selecting appropriate dietary strategies according to metabolic phenotype [102]. Emerging technologies involving metabolomics, lipidomics, proteomics, and microbiome sequencing are expected to refine these approaches even further. However, most proposed biomarkers remain investigational, and robust clinical validation is still lacking [35].

Importantly, the phenotype-based associations proposed here should be interpreted as a framework for hypothesis generation and clinical individualization rather than as validated treatment rules. No biomarker has yet been sufficiently validated to prospectively determine the optimal dietary intervention for a specific OSA phenotype. Table 4 therefore integrates candidate phenotypes, measurable characteristics, potential nutritional strategies, available evidence, and major uncertainties to illustrate how precision nutrition could be operationalized in future clinical research.

### 8.6. Artificial Intelligence and Digital Nutrition

Rapid advances in digital health technologies are transforming nutritional care. Wearable devices, continuous glucose monitoring systems, smartphone-based dietary assessment, remote monitoring platforms, and artificial intelligence (AI) algorithms increasingly allow real-time evaluation of dietary intake, physical activity, sleep behavior, and metabolic responses. Machine learning models integrating clinical, behavioral, nutritional, microbiome, and omics data may eventually predict which patients are most likely to respond to specific dietary interventions or pharmacological therapies. These technologies could facilitate highly individualized nutritional prescriptions while improving adherence through continuous monitoring and personalized feedback [1]. Although these approaches remain in early stages of implementation, they represent one of the most promising frontiers in precision medicine for OSA.

### 8.7. Future Perspective

Precision nutrition represents a natural evolution of nutritional therapy in OSA. Rather than focusing exclusively on caloric restriction and weight loss, future dietary interventions are likely to incorporate multidimensional biological information, including metabolic phenotype, inflammatory profile, gut microbiota composition, body composition, and behavioral characteristics. The integration of nutritional science with incretin-based pharmacotherapy, multi-omics technologies, digital health, and artificial intelligence offers unprecedented opportunities to individualize treatment and optimize long-term outcomes. Nevertheless, large prospective randomized trials are still needed to validate precision nutrition strategies specifically in patients with OSA and to determine their incremental benefit beyond conventional lifestyle interventions.

Although precision nutrition and incretin-based therapies represent exciting advances, translating these concepts into routine clinical practice remains challenging. Healthcare professionals require practical frameworks that integrate nutritional assessment, patient phenotyping, behavioral counseling, pharmacological therapy, and multidisciplinary care. Therefore, the following section discusses the current clinical implications of nutritional management in OSA and proposes practical recommendations based on the best available evidence.

## 9. Clinical Implications: Integrating Nutrition into the Multidisciplinary Management of Obstructive Sleep Apnea

The management of OSA has traditionally focused on alleviating upper airway obstruction through CPAP, oral appliances, upper airway surgery, and positional therapy. However, growing recognition of OSA as a systemic cardiometabolic disorder underscores the need for comprehensive treatment strategies that address the underlying biological mechanisms contributing to disease progression [2]. Nutrition should therefore be regarded as a core component of multidisciplinary OSA management rather than merely an adjunctive strategy for weight reduction [102].

### 9.1. Nutritional Assessment Should Become Routine in OSA Care

A comprehensive nutritional assessment should be incorporated into the initial evaluation of all patients with OSA. While BMI remains widely used in clinical practice, it provides limited information regarding body composition and metabolic health [2]. Assessment should therefore include waist circumference, neck circumference, visceral adiposity, dietary quality, eating behaviors, meal timing, alcohol consumption, physical activity, and the presence of obesity-related comorbidities. Whenever feasible, body composition analysis using dual-energy X-ray absorptiometry (DXA), air displacement plethysmography, or validated bioelectrical impedance analysis can provide clinically relevant information regarding fat distribution and skeletal muscle mass. This is particularly important because sarcopenic obesity is increasingly recognized as an adverse metabolic phenotype associated with poorer cardiometabolic outcomes [104]. Dietary assessment should also identify excessive intake of ultra-processed foods, inadequate dietary fiber consumption, poor adherence to Mediterranean-style dietary patterns, irregular meal timing, and behaviors such as emotional eating or late-night eating, all of which may influence both metabolic health and sleep quality [102].

### 9.2. Individualized Nutritional Interventions

Current evidence suggests that nutritional interventions should be individualized according to each patient’s clinical characteristics rather than applying identical recommendations to all individuals with OSA [104]. For patients with obesity-related OSA, structured weight-loss programs remain the cornerstone of nutritional management. These interventions should prioritize sustainable reductions in body weight while preserving lean body mass through adequate protein intake and regular resistance exercise [105]. In patients with prominent metabolic dysfunction, including insulin resistance, type 2 diabetes mellitus, metabolic dysfunction-associated MASLD, or severe visceral obesity, nutritional interventions should emphasize improved glycemic control, increased dietary fiber intake, reduced consumption of refined carbohydrates and ultra-processed foods, and adoption of Mediterranean or DASH dietary patterns. Conversely, in non-obese individuals with anatomically driven OSA, the primary objective may not be weight loss but rather optimization of overall dietary quality, reduction in systemic inflammation, improvement of endothelial function, and prevention of long-term cardiometabolic complications [103,104,105,106,107,108,109,110]. This individualized approach aligns closely with current concepts of precision medicine and reinforces the importance of multidisciplinary care involving sleep physicians, dietitians, endocrinologists, exercise physiologists, and behavioral health professionals.

### 9.3. Combining Nutritional Therapy with CPAP and Pharmacological Treatment

Nutritional interventions should complement rather than replace established OSA therapies. Although CPAP effectively reduces respiratory events, its effects on obesity, insulin resistance, systemic inflammation, and cardiovascular risk are often modest when used in isolation [100,119]. Lifestyle interventions combining dietary modification, structured exercise, and CPAP produce greater improvements in body composition, blood pressure, glycemic control, inflammatory biomarkers, and quality of life than CPAP alone [100]. Similarly, patients receiving incretin-based therapies benefit substantially from concurrent nutritional counseling aimed at maintaining dietary quality, preserving skeletal muscle mass, and supporting long-term weight maintenance. This integrated therapeutic model recognizes that mechanical treatment of upper airway obstruction and metabolic treatment of obesity are complementary components of comprehensive OSA care [119].

### 9.4. Monitoring Treatment Response

Evaluation of nutritional interventions should extend beyond changes in body weight alone. Although reductions in BMI remain clinically important, they may underestimate improvements in metabolic health. Treatment monitoring should ideally include changes in: AHI; oxygen desaturation index (ODI); time spent with oxygen saturation below 90% (T90); waist and neck circumference; body composition; blood pressure; glycemic control; lipid profile; CRP; health-related quality of life; daytime sleepiness; dietary adherence. Assessment of these multidimensional outcomes provides a more comprehensive evaluation of therapeutic effectiveness and aligns with current precision medicine principles.

### 9.5. Current Challenges

Despite growing evidence supporting nutritional interventions, several barriers limit their incorporation into routine OSA care. Many sleep clinics lack access to dietitians with expertise in sleep medicine, and nutritional counseling is frequently restricted to general recommendations for weight loss [108,109]. Patient adherence also remains challenging because untreated OSA is commonly associated with excessive daytime sleepiness, fatigue, depression, impaired executive function, and reduced motivation for behavioral change [110]. Consequently, successful nutritional management often requires motivational interviewing, behavioral counseling, long-term follow-up, and multidisciplinary support. Another important challenge is the limited availability of randomized controlled trials specifically evaluating dietary interventions in OSA [111]. Most current recommendations continue to be extrapolated from studies involving obesity, diabetes, hypertension, or cardiovascular disease, emphasizing the need for disease-specific nutritional research [108].

### 9.6. Proposed Clinical Framework

Based on the currently available evidence, nutritional management of OSA should follow several fundamental principles:

1. Perform comprehensive nutritional assessment, including body composition, dietary quality, metabolic risk factors, and eating behaviors.
2. Prioritize healthy dietary patterns, particularly Mediterranean and DASH diets, instead of focusing exclusively on caloric restriction.
3. Reduce consumption of ultra-processed foods, sugar-sweetened beverages, and saturated fats.
4. Increase dietary fiber intake through fruits, vegetables, legumes, whole grains, and nuts to improve gut microbiota composition and metabolic health.
5. Individualize nutritional therapy according to obesity phenotype, metabolic profile, comorbidities, and patient preferences.
6. Integrate nutrition with CPAP, exercise, and pharmacological therapy, particularly incretin-based medications when indicated.
7. Monitor multidimensional clinical outcomes, including respiratory, metabolic, inflammatory, and behavioral parameters.

Although these recommendations are supported by growing biological plausibility and accumulating clinical evidence, further high-quality randomized clinical trials are still needed to establish disease-specific nutritional guidelines for OSA. The integration of anthropometric, metabolic, inflammatory, behavioral, and sleep-related characteristics provides the basis for precision nutrition in OSA. Rather than adopting a one-size-fits-all dietary approach, nutritional management should be tailored according to the patient’s predominant phenotype, cardiometabolic profile, gut microbiota status, circadian behavior, and treatment goals. Figure 4 summarizes conceptual framework for implementing a nutrition approach in adults with OSA (Figure 4).

Over the past decade, substantial progress has been made in understanding the complex interactions among nutrition, metabolism, inflammation, gut microbiota, and obstructive sleep apnea. Nevertheless, important knowledge gaps remain regarding the optimal dietary strategies for different OSA phenotypes, the role of precision nutrition, and the integration of nutritional interventions with emerging pharmacological therapies. Addressing these challenges will require multidisciplinary research combining sleep medicine, nutritional science, metabolomics, microbiome research, and digital health technologies.

## 10. Future Directions

The growing recognition of OSA as a complex, heterogeneous cardiometabolic disorder has created new opportunities for nutritional research. While current evidence strongly supports the role of healthy dietary patterns in improving obesity-related outcomes and reducing cardiometabolic risk, many fundamental questions regarding the optimal nutritional management of OSA remain unanswered. Addressing these gaps will require a transition from conventional dietary recommendations toward mechanism-based, individualized interventions supported by advances in systems biology, digital health, and precision medicine.

One of the highest research priorities is the identification of nutritional strategies tailored to specific OSA phenotypes and endotypes. Most available dietary interventions have been evaluated in heterogeneous populations, limiting the ability to determine whether particular subgroups, such as patients with severe visceral obesity, insulin resistance, REM-predominant OSA, positional OSA, or prominent inflammatory profiles, respond differently to specific dietary approaches. Future randomized clinical trials should therefore incorporate comprehensive phenotyping, including body composition, metabolic status, inflammatory biomarkers, and physiological endotypes, to facilitate personalized nutritional recommendations.

Another important area of investigation is the integration of multi-omics technologies into nutritional research. Advances in genomics, epigenomics, transcriptomics, proteomics, metabolomics, lipidomics, and microbiomics provide unprecedented opportunities to characterize the biological heterogeneity of OSA. Combining these technologies may enable the identification of biomarkers capable of predicting dietary responsiveness, monitoring treatment efficacy, and identifying individuals most likely to benefit from specific nutritional interventions. Such approaches are expected to move nutritional care from population-based recommendations toward truly individualized treatment strategies.

The gut microbiota represents another rapidly evolving field with substantial therapeutic potential. Although experimental evidence strongly supports a mechanistic role for gut dysbiosis in intermittent hypoxia-induced metabolic dysfunction, prospective human studies remain limited. Future investigations should evaluate whether microbiome-targeted interventions, including high-fiber dietary patterns, prebiotics, probiotics, synbiotics, postbiotics, and personalized microbiota-based nutrition, can improve respiratory outcomes in addition to metabolic health. Longitudinal studies combining microbiome sequencing with metabolomic analyses may further clarify the causal relationships between intestinal microbial communities and OSA progression.

Chrononutrition also deserves greater attention. Most dietary studies have focused on caloric intake and macronutrient composition while largely overlooking meal timing, circadian alignment, and eating behaviors. Randomized controlled trials evaluating time-restricted eating, early time-restricted feeding, meal distribution, and circadian-based dietary interventions specifically in patients with OSA are urgently needed. Such studies should incorporate objective sleep measurements, circadian biomarkers, and long-term cardiometabolic outcomes to better understand the interaction between nutritional timing and sleep physiology [40].

The rapid development of incretin-based pharmacotherapies has also transformed the landscape of obesity treatment and OSA management. Future research should determine how nutritional interventions can optimize treatment with GLP-1RAs, dual GIP/GLP-1 receptor agonists, and emerging multi-agonist therapies. Particular attention should be given to preserving lean body mass during substantial weight loss, maintaining adequate micronutrient intake, and identifying dietary strategies that maximize long-term treatment adherence and metabolic benefits.

AI and digital health technologies are expected to play an increasingly important role in precision nutrition. Wearable devices capable of continuously monitoring sleep, physical activity, heart rate, glucose variability, and dietary behaviors may facilitate real-time personalization of nutritional recommendations. Machine learning algorithms integrating clinical, behavioral, imaging, and multi-omics data could support predictive models that identify patients most likely to respond to specific dietary interventions, thereby improving treatment efficiency and reducing healthcare costs. Importantly, future clinical trials should move beyond evaluating reductions in body weight and AHI alone. Comprehensive assessment of treatment efficacy should include inflammatory biomarkers, endothelial function, vascular stiffness, autonomic regulation, body composition, gut microbiota composition, health-related quality of life, cognitive performance, and long-term cardiovascular outcomes. Such multidimensional endpoints will provide a more complete understanding of the biological effects of nutritional interventions.

Finally, the development of evidence-based nutritional guidelines specifically for OSA should become a priority for international sleep and nutrition societies. Current recommendations are largely extrapolated from obesity and cardiovascular disease guidelines, despite the unique pathophysiological characteristics of OSA. High-quality multicenter randomized controlled trials with standardized dietary interventions, adequate follow-up, and objective sleep measurements will be essential to establish disease-specific nutritional recommendations and strengthen the role of nutrition within multidisciplinary sleep medicine.

## 11. Conclusions

Obstructive sleep apnea is a heterogeneous disorder in which upper-airway obstruction interacts with obesity, metabolic dysfunction, inflammation, oxidative stress, endothelial alterations, circadian disruption, and other systemic processes. Within this complex framework, nutrition represents an important component of comprehensive OSA management, with potential effects extending beyond body-weight reduction. Current evidence supports the prioritization of healthy dietary patterns characterized by minimally processed foods, dietary fiber, unsaturated fats, fruits, vegetables, legumes, whole grains, nuts, and other nutrient-dense foods. Mediterranean- and DASH-oriented approaches appear particularly relevant for cardiometabolic risk management, although the number of OSA-specific randomized trials remains limited. Evidence concerning individual nutrients, bioactive compounds, chrononutrition, gut microbiota modulation, and other emerging approaches is predominantly mechanistic, observational, indirect, or based on small clinical studies. These areas therefore require further validation before they can be considered disease-specific nutritional therapies.

The heterogeneity of OSA provides a rationale for moving toward precision nutrition, in which nutritional strategies are considered in relation to body composition, metabolic phenotype, inflammatory profile, behavioral characteristics, circadian factors, and potentially validated biomarkers. However, no biomarker-guided nutritional algorithm has yet been sufficiently validated to determine the optimal dietary intervention for a specific OSA phenotype. Incretin-based pharmacotherapies further reinforce the importance of integrating nutritional management with pharmacological treatment, particularly for patients with obesity and metabolic dysfunction. Nutritional counseling may support dietary quality, preservation of lean mass, gastrointestinal tolerance, and long-term weight management during pharmacotherapy. Nevertheless, the optimal combination of pharmacological and nutritional interventions remains to be established.

Future research should prioritize adequately powered, prospective, OSA-specific randomized clinical trials using standardized dietary interventions and objective respiratory outcomes. Studies should also incorporate body composition, metabolic biomarkers, inflammatory markers, circadian variables, and, where appropriate, microbiome and multi-omics measures. Such studies will be necessary to determine whether phenotype-guided nutritional interventions provide benefits beyond conventional lifestyle and weight-management strategies. Overall, nutrition should be integrated into multidisciplinary OSA care as a complementary therapeutic component rather than as a replacement for established treatments such as CPAP. The transition from generalized dietary advice toward evidence-based precision nutrition represents a promising direction, but its clinical implementation should remain proportional to the current level of evidence.

## Figures and Tables

**Figure 1:**
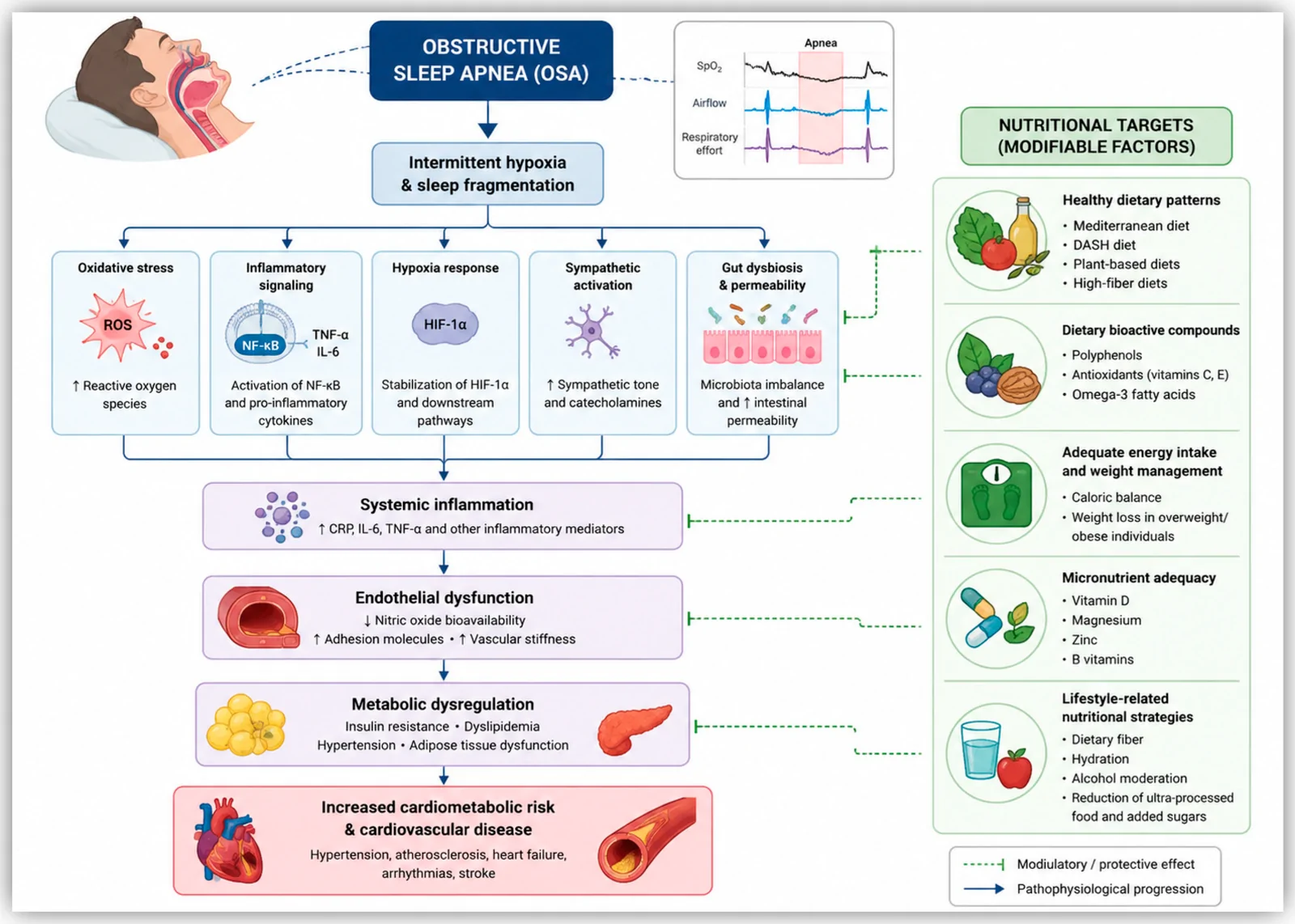
Biological mechanisms linking nutrition and obstructive sleep apnea (OSA). This schematic summarizes how nutrition interacts with key pathophysiological pathways in OSA. Recurrent intermittent hypoxia and disrupted sleep continuity activate oxidative stress, inflammatory signaling, hypoxia-responsive pathways, sympathetic overactivity, and gut dysbiosis, contributing to systemic inflammation, endothelial dysfunction, and metabolic impairment. Nutritional strategies, such as Mediterranean, DASH, plant-based, and high-fiber dietary patterns, along with adequate intake of polyphenols, antioxidants, omega-3 fatty acids, and appropriate weight management, may modulate these processes by improving inflammation, oxidative balance, gut microbiota composition, endothelial function, and metabolic homeostasis. Blue arrows depict disease progression, whereas green dashed lines represent the modulatory effects of nutritional interventions. Abbreviations: CRP, C-reactive protein; DASH, Dietary Approaches to Stop Hypertension; GLP-1, glucagon-like peptide-1; GIP, glucose-dependent insulinotropic polypeptide; HIF-1α, hypoxia-inducible factor-1 alpha; IL-6, interleukin-6; NF-κB, nuclear factor kappa B; OSA, obstructive sleep apnea; ROS, reactive oxygen species; TNF-α, tumor necrosis factor alpha. An upward arrow indicates an increase, while a downward arrow indicates a decrease.

**Figure 2:**
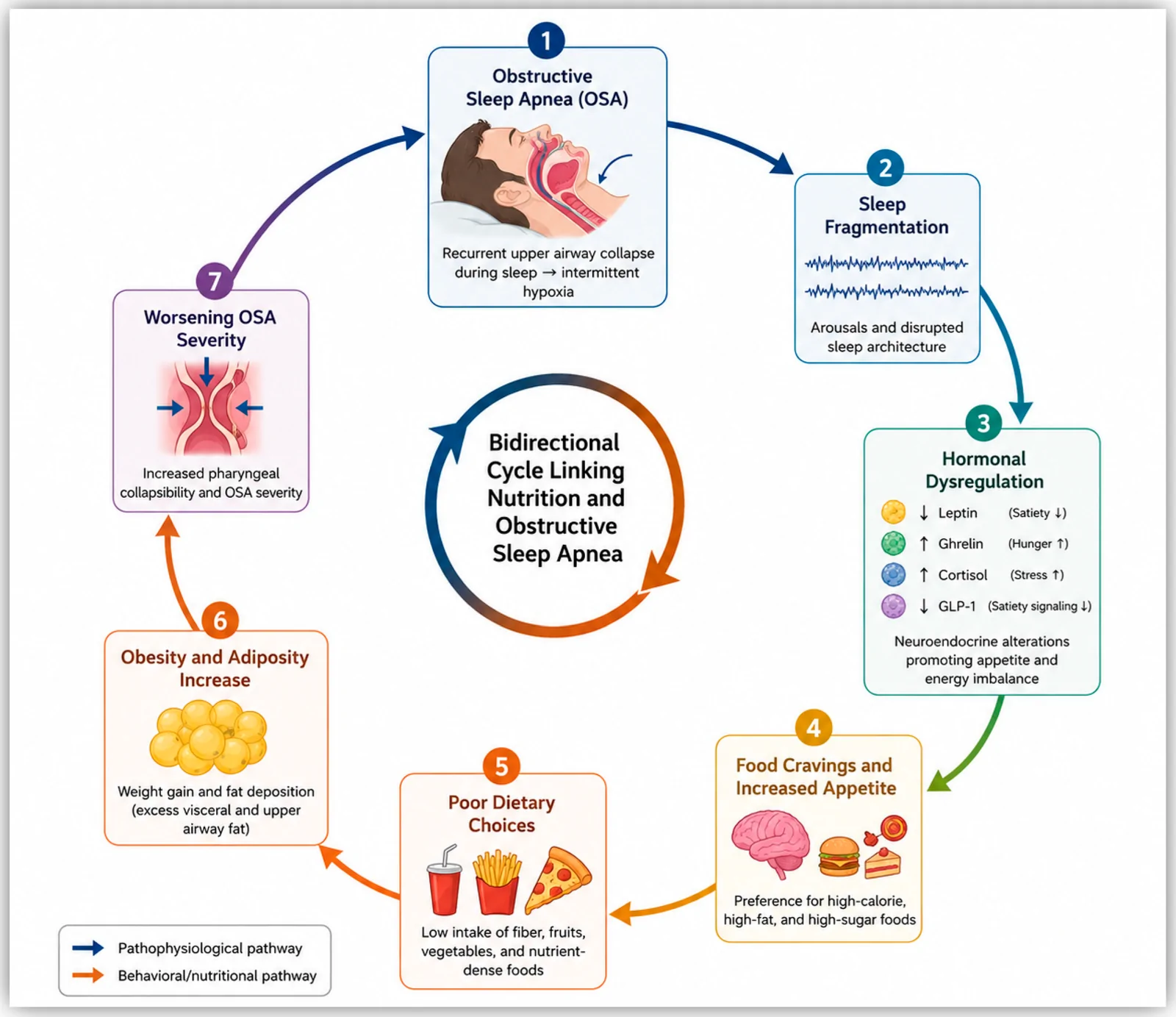
The bidirectional relationship between nutrition and obstructive sleep apnea (OSA). This figure illustrates the self-reinforcing cycle linking OSA and nutrition. Recurrent intermittent hypoxia and disrupted sleep continuity lead to neuroendocrine alterations, including reduced leptin sensitivity and GLP-1 signaling, alongside increased ghrelin and cortisol levels. These changes heighten appetite, food cravings, and preference for energy-dense, ultra-processed foods. Poor dietary quality promotes positive energy balance, weight gain, and visceral and upper-airway fat deposition, increasing pharyngeal collapsibility and worsening OSA severity. This cycle highlights the reciprocal interactions among sleep-disordered breathing, hormonal regulation, eating behavior, and obesity, underscoring multiple opportunities for nutritional and lifestyle interventions to interrupt disease progression. Abbreviations: GLP-1, glucagon-like peptide-1; OSA, obstructive sleep apnea. An upward arrow indicates an increase, while a downward arrow indicates a decrease.

**Figure 3:**
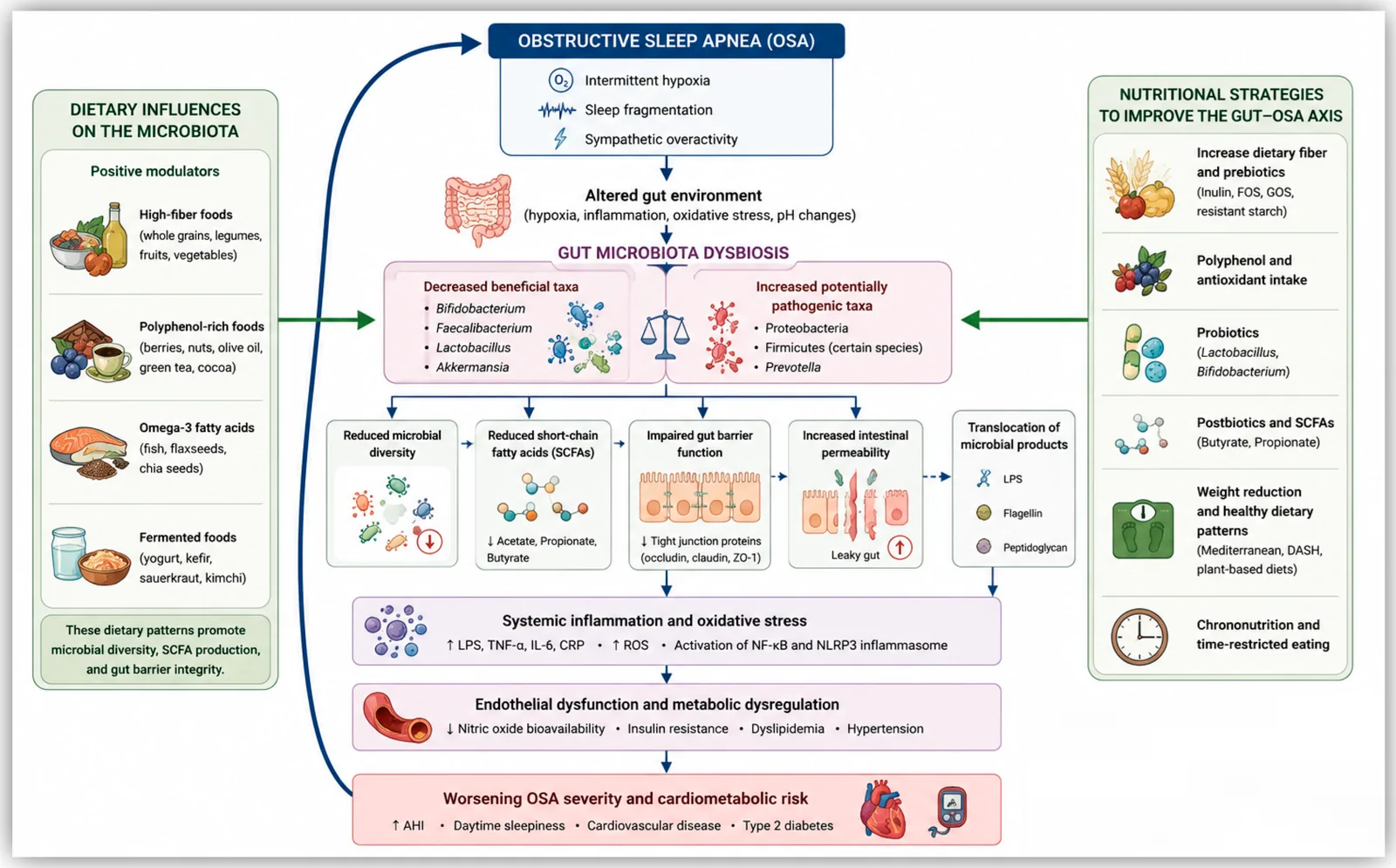
The gut microbiota–obstructive sleep apnea (OSA) axis: mechanisms and nutritional modulation. This figure summarizes how OSA alters the gut microbiota and how nutrition may modulate these effects. Intermittent hypoxia, disrupted sleep continuity, and sympathetic overactivity promote dysbiosis, reduced microbial diversity, and lower short-chain fatty acid (SCFA) production. These changes impair intestinal barrier integrity and increase permeability, allowing translocation of microbial products such as lipopolysaccharide (LPS), which activate inflammatory pathways including NF-κB and the NLRP3 inflammasome. The resulting oxidative stress, systemic inflammation, endothelial dysfunction, and metabolic impairment contribute to increased cardiometabolic risk and worsen OSA severity. High-fiber diets, prebiotics, probiotics, polyphenol-rich foods, omega-3 fatty acids, and Mediterranean or DASH dietary patterns may help restore microbial diversity, enhance SCFA production, strengthen the intestinal barrier, and reduce inflammation. Abbreviations: AHI, apnea–hypopnea index; CRP, C-reactive protein; DASH, Dietary Approaches to Stop Hypertension; IL-6, interleukin-6; LPS, lipopolysaccharide; NF-κB, nuclear factor kappa B; NLRP3, NOD-like receptor family pyrin domain-containing 3; OSA, obstructive sleep apnea; ROS, reactive oxygen species; SCFAs, short-chain fatty acids; TNF-α, tumor necrosis factor alpha; ZO-1, zonula occludens-1. An upward arrow indicates an increase, while a downward arrow indicates a decrease.

**Figure 4:**
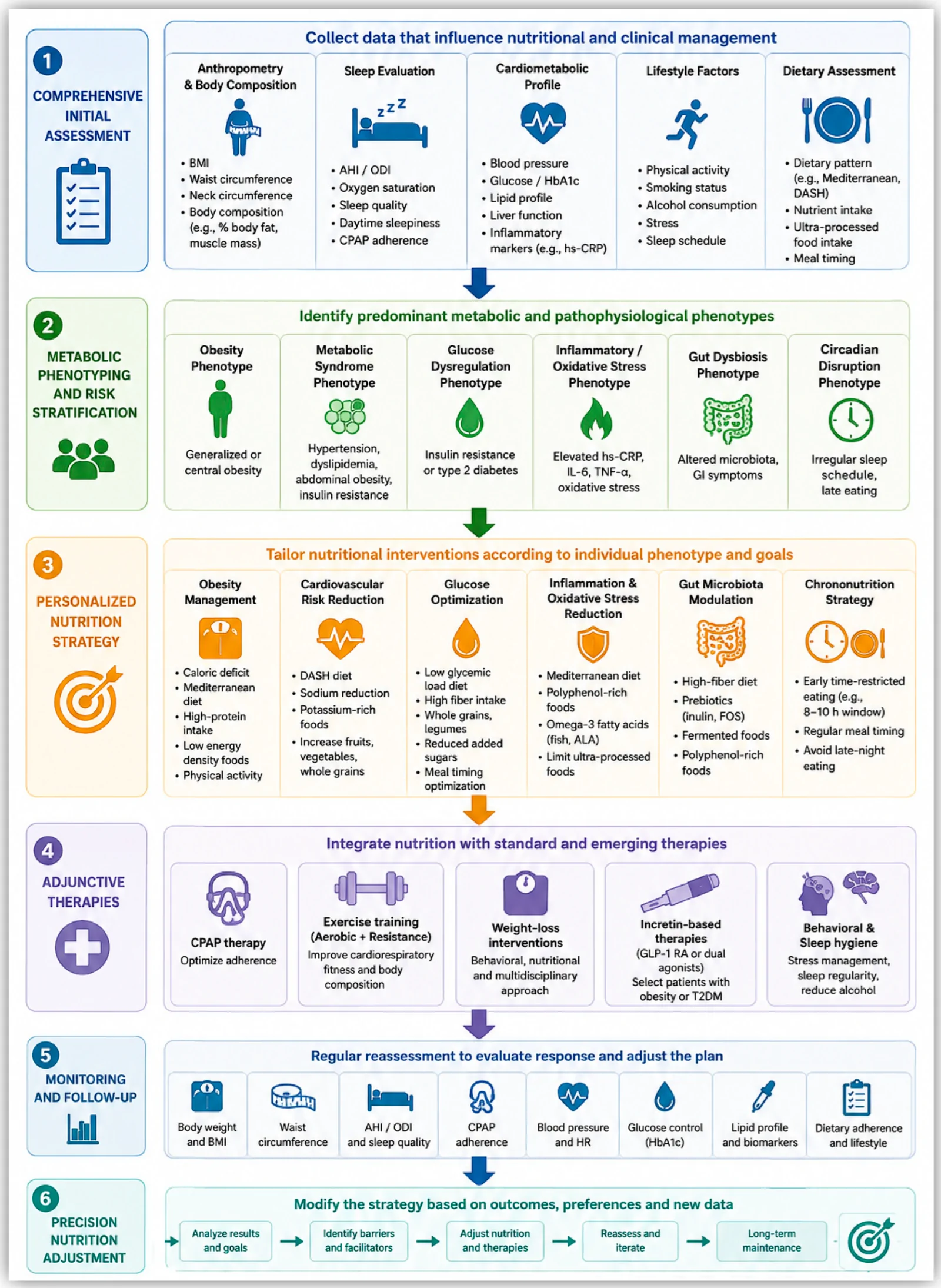
Conceptual nutrition framework for the nutritional management of adults with obstructive sleep apnea (OSA). This figure outlines a precision-nutrition approach for adults with OSA. Clinical evaluation should include anthropometry, sleep characteristics, cardiometabolic status, lifestyle, and dietary habits. Metabolic phenotypes, such as obesity, metabolic syndrome, insulin resistance, gut dysbiosis, and circadian disruption, can guide individualized strategies including Mediterranean or DASH dietary patterns, chrononutrition, anti-inflammatory diets, and gut-microbiota-targeted interventions. Nutritional therapy should be integrated with standard OSA treatments such as CPAP, physical exercise, and, when appropriate, incretin-based pharmacotherapy. Ongoing reassessment allows adjustment of dietary interventions according to changes in metabolic profile and sleep outcomes. Abbreviations: AHI, apnea–hypopnea index; BMI, body mass index; CPAP, continuous positive airway pressure; DASH, Dietary Approaches to Stop Hypertension; GI, gastrointestinal; GLP-1, glucagon-like peptide-1; HbA1c, glycated hemoglobin; HDL-C, high-density lipoprotein cholesterol; hs-CRP, high-sensitivity C-reactive protein; IL-6, interleukin-6; LDL-C, low-density lipoprotein cholesterol; NAFLD, nonalcoholic fatty liver disease; ODI, oxygen desaturation index; OSA, obstructive sleep apnea; TNF-α, tumor necrosis factor-alpha; T2DM, type 2 diabetes mellitus.

**Table 1:** Biological mechanisms linking nutrition to obstructive sleep apnea.

Physiological Domain	Mechanistic Link to OSA	Nutritional Factors/Interventions	Potential Clinical Relevance	Evidence Type
Adiposity and airway anatomy [8,10,33].	Visceral and upper-airway adiposity increase pharyngeal collapsibility and reduce respiratory reserve	Calorie-controlled dietary patterns; Mediterranean/DASH diets; weight management	Reduction in body weight and visceral adiposity may reduce OSA severity	OSA clinical studies; indirect evidence
Inflammation [2,8,33,38,74,85].	Intermittent hypoxia and adiposity promote NF-κB signaling and inflammatory cytokines	Mediterranean/plant-based diets; fiber; polyphenol-rich foods	May improve systemic inflammatory and cardiometabolic profiles	Mechanistic + observational + clinical evidence
Oxidative stress [2,8,33,38,74,85].	Hypoxia–reoxygenation increases ROS and reduces nitric oxide bioavailability	Polyphenols; antioxidant-rich foods; omega-3 fatty acids	Potential improvement in oxidative and endothelial function	Mechanistic + indirect human evidence
Insulin resistance and metabolic dysfunction [32,42,46,65,80].	Intermittent hypoxia and obesity impair insulin signaling and glucose metabolism	Mediterranean/DASH diets; dietary fiber; weight loss	May improve metabolic health and indirectly reduce OSA burden	OSA and obesity clinical evidence
Gut microbiota [38,41,71].	Dysbiosis may contribute to inflammation, metabolic dysfunction, and endotoxemia	Fiber; prebiotics; probiotics; minimally processed diets	Potential modulation of metabolic and inflammatory pathways	Preclinical + limited human evidence
Endothelial dysfunction [28,49,66,74,78,79].	Oxidative stress and inflammation reduce NO availability and impair vascular function	Mediterranean/DASH diets; omega-3-rich foods; nitrate-rich vegetables	Potential improvement in vascular and cardiometabolic risk	Mechanistic + clinical/indirect evidence
Circadian regulation [2,33,38,78].	Irregular meal timing and nighttime eating may disrupt metabolic and circadian signaling	Regular meal timing; avoidance of large late-night meals; time-restricted eating	Potential improvement in metabolic and sleep-related outcomes	Indirect human + limited OSA-specific evidence
Sleep-related neuroendocrine regulation [2,4,33,38,40,41,78].	Leptin, ghrelin, orexin, tryptophan/serotonin/melatonin pathways link nutrition with sleep regulation	Adequate protein/tryptophan; vitamin B6-containing foods; regular feeding schedules	Potential support for sleep quality and circadian alignment	Mechanistic + indirect human evidence

Abbreviations: CRP, C-reactive protein; IL-6, interleukin-6; NF-κB, nuclear factor-kappa B; OSA, obstructive sleep apnea; ROS, reactive oxygen species; TNF-α, tumor necrosis factor-alpha.

**Table 2:** Summary of clinical evidence for dietary patterns in obstructive sleep apnea.

Dietary Pattern	Main Mechanism/Target	Evidence Directly Available in OSA	Main Limitation
Mediterranean [2,5,17].	Cardiometabolic and inflammatory pathways	OSA-specific clinical and observational evidence	Few controlled OSA-specific trials
DASH [40,42].	Blood pressure and cardiometabolic regulation	OSA-specific and indirect cardiometabolic evidence	Limited OSA-specific intervention studies
Plant-based [54,56].	Dietary quality and weight/metabolic regulation	Mainly observational and indirect evidence	Predominantly non-randomized evidence
Low-carbohydrate [86,87].	Weight and metabolic regulation	OSA-specific and indirect evidence	Heterogeneous dietary protocols and follow-up
Ketogenic [5,104].	Weight and metabolic regulation	Limited OSA-specific clinical evidence	Small studies and uncertainty regarding long-term applicability
Time-restricted eating [5,17,40,42,54,56,86,87,104,108,109,110,111,112,113,114,115].	Circadian and metabolic regulation	Preliminary human and indirect evidence	Few OSA-specific intervention studies

Abbreviations: AHI, apnea–hypopnea index; DASH, Dietary Approaches to Stop Hypertension; OSA, obstructive sleep apnea; RCT, randomized controlled trial.

**Table 3:** Micronutrients and bioactive compounds investigated in obstructive sleep apnea.

Compound	Main Biological Pathway	Evidence in OSA	Practical Interpretation
Vitamin D [2,4,19,23,24,25].	Immune regulation, skeletal muscle function, inflammation	Observational human evidence; intervention evidence remains heterogeneous	Correct documented deficiency; routine supplementation specifically for OSA is not established
Omega-3 fatty acids [2,19,47,51,52,99].	Inflammation, endothelial and autonomic regulation	Mainly observational and indirect evidence; few OSA-specific intervention studies	Prefer dietary sources such as oily fish, nuts, and seeds
Dietary fiber [105,109,110,111,112,113,114,115,116,117,118].	Gut microbiota, SCFA production, metabolic regulation	Indirect human and mechanistic evidence; limited OSA-specific intervention data	Encourage fiber-rich foods as part of healthy dietary patterns
Polyphenols [2,51,52,99,105,109,110,111,112,113,114,115,116,117,118].	Antioxidant, anti-inflammatory, endothelial and microbiota-related pathways	Mechanistic and indirect human evidence	Prefer food-based sources; OSA-specific respiratory efficacy remains uncertain
Magnesium [2,4,19,23,24,25,27,47,51].	Neuromuscular and metabolic regulation	Observational and indirect evidence	Ensure adequate dietary intake; supplementation should be clinically indicated
Melatonin [2,4,19,23,24,25,27,47,51,52,99,105,109,110,111,112,113,114,115,116,117,118].	Circadian regulation and antioxidant pathways	Mechanistic and limited clinical evidence in OSA	Not recommended as routine OSA therapy based solely on current evidence
Vitamin B6 [2,4,19,25,27].	Tryptophan metabolism and neurotransmitter synthesis	Mechanistic and nutritional evidence; limited OSA-specific clinical evidence	Ensure adequate intake; supplementation should be based on nutritional indication
Iron [2,4,19,107].	Oxygen transport, mitochondrial and neuromuscular function	Indirect and nutritional evidence; OSA-specific evidence is limited	Assess and correct documented deficiency
N-acetylcysteine [2,4,19,85].	Glutathione synthesis and antioxidant pathways	Preliminary mechanistic and clinical evidence; few OSA-specific trials	Investigational; insufficient evidence for routine OSA use

Abbreviations: DHA, docosahexaenoic acid; EPA, eicosapentaenoic acid; GLP-1, glucagon-like peptide-1; OSA, obstructive sleep apnea; SCFA, short-chain fatty acid.

**Table 4:** Conceptual precision nutrition framework for adults with obstructive sleep apnea.

OSA Phenotype/ Endotype	Candidate Biomarkers/ Characteristics	Potential Nutritional Strategy	Main Therapeutic Target	Evidence Basis	Main Uncertainty
Obesity-related OSA with visceral adiposity [5,79,80,81,82,83,84,85,86,87,88,89,90,91].	BMI, waist circumference, body composition, visceral adipose tissue	Energy-controlled Mediterranean or DASH dietary pattern	Weight reduction and metabolic improvement	OSA-specific and indirect clinical evidence	Independent contribution of diet beyond weight loss
OSA with insulin resistance/metabolic dysfunction [5,79,80,81,82,83,84,85,86,87,88,89,90,91,95,96,97,98,99].	Fasting insulin, HOMA-IR, HbA1c, triglycerides, HDL-C	Mediterranean/DASH pattern and individualized energy restriction	Insulin sensitivity and cardiometabolic risk	OSA and indirect metabolic evidence	Phenotype-specific respiratory benefit
OSA with systemic inflammatory profile [5,79,80,81,82,83,84,85,86,87,88,89,90,91,95,96,97,98,99,102,104,105,119].	CRP, IL-6, TNF-α, adipokines	Fiber-rich, minimally processed, plant-rich dietary pattern	Inflammatory regulation	Mechanistic and observational evidence	Effect on objective respiratory outcomes
OSA with circadian or behavioral misalignment [79,80,81,82,83,84,85,86,87,88,89,90,91,95,96,97,98,99,102,104,105,119].	Sleep timing, meal timing, light exposure, sleep regularity	Regular daytime-centered meals and avoidance of large late meals; circadian alignment strategies	Circadian and metabolic regulation	Mechanistic, observational, and preliminary intervention evidence	Lack of standardized OSA-specific protocols
OSA with suspected gut dysbiosis [5,79,80,81,82,83,84,85,86,87,88,89,90,91,102,104,105,119].	Microbiome composition, SCFAs, metabolic markers	Increased dietary fiber and minimally processed plant foods	Gut barrier and metabolic regulation	Mechanistic, animal, and observational human evidence	No validated microbiome-guided treatment algorithm
OSA with normal body weight and predominantly anatomical susceptibility [5,79,80,81,82,83,84,85,86,87,88,89,90,91,95,96,97,98,99,102,104,105,119].	Body composition, craniofacial features, respiratory phenotype	Avoid unnecessary caloric restriction; focus on overall dietary quality	Cardiometabolic health and treatment support	Limited direct evidence	Nutritional effect on anatomical OSA mechanisms remains uncertain
OSA treated with incretin-based pharmacotherapy [95,96,97,98,99].	Body weight, body composition, glycemic markers	Nutritional counseling emphasizing adequate protein, dietary quality, and preservation of lean mass	Weight loss, metabolic health, treatment adherence	OSA-specific pharmacological evidence plus indirect nutritional evidence	Optimal combined nutritional protocols remain undefined

Abbreviations: OSA: Obstructive Sleep Apnea; BMI: Body Mass Index; CRP: C-reactive protein; IL-6: Interleukin-6; TNF-α: Tumor Necrosis Factor alpha; HDL-C: High-density lipoprotein cholesterol; HOMA-IR: Homeostatic Model Assessment of Insulin Resistance; HbA1c: Hemoglobin A1c; SCFAs: short-chain fatty acids; DASH: Dietary Approaches to Stop Hypertension.

## Data Availability

No new data were created or analyzed in this study. Data sharing is not applicable to this article.

## Supplementary Materials

The following supporting information can be downloaded at: https://www.mdpi.com/article/10.3390/clockssleep8040058/s1.

## Abbreviations

The following abbreviations are used in this manuscript:

AHI	Apnea–Hypopnea Index
AI	Artificial Intelligence
BMI	Body Mass Index
CPAP	Continuous Positive Airway Pressure
CRP	C-Reactive Protein
DASH	Dietary Approaches to Stop Hypertension
DHA	Docosahexaenoic Acid
DXA	Dual-Energy X-ray Absorptiometry
EPA	Eicosapentaenoic Acid
GLP-1	Glucagon-Like Peptide-1
GLP-1RAs	Glucagon-Like Peptide-1 Receptor Agonist
GIP	Glucose-Dependent Insulinotropic Polypeptide
IL	Interleukin
LPS	Lipopolysaccharide
MASLD	Metabolic Dysfunction-Associated Steatotic Liver Disease
NF-κB	Nuclear Factor-Kappa B
Nrf2	Nuclear Factor Erythroid 2-Related Factor 2
OSA	Obstructive Sleep Apnea
PUFAs	Polyunsaturated Fatty Acids
RCT	Randomized Controlled Trial
REM	Rapid Eye Movement
ROS	Reactive Oxygen Species
SCFAs	Short-Chain Fatty Acids
TLR4	Toll-Like Receptor 4
TNF-α	Tumor Necrosis Factor-Alpha
