# Supplementary material for: Dietary Approaches to Obstructive Sleep Apnea: Translating Mechanistic Pathways into Clinical Practice
Source: Clocks Sleep. 2026 Sep 22;8(4):58. doi: 10.3390/clockssleep8040058 (PMC13692405; doi:10.3390/clockssleep8040058)
Supplement: Supplementary file 1 [file clockssleep-08-00058-s001.zip › clockssleep-4493518-supplementary.pdf]

Supplementary Materials

Complete database-specific search strategies

The literature search was conducted in PubMed/MEDLINE, Scopus, and Web of Science Core Collection. Search strategies were developed using three conceptual domains: (1) obstructive sleep apnea, (2) nutrition and dietary interventions, and (3) biological mechanisms and emerging precision-nutrition approaches. Database-specific syntax and search fields were adapted according to the indexing structure of each database. Searches covered publications published from January 2000 through June 2026 and were restricted to publications in English, Portuguese, or Spanish.

1. PubMed/MEDLINE

Search strategy:

```
(
  "Obstructive Sleep Apnea"[Mesh]
  OR "obstructive sleep apnea"[Title/Abstract]
  OR "obstructive sleep apnoea"[Title/Abstract]
  OR OSA[Title/Abstract]
  OR "sleep-disordered breathing"[Title/Abstract]
)
AND
(
  "Nutrition Therapy"[Mesh]
  OR "Diet"[Mesh]
  OR nutrition[Title/Abstract]
  OR diet*[Title/Abstract]
  OR "dietary pattern*"[Title/Abstract]
  OR "Mediterranean diet"[Title/Abstract]
  OR "DASH diet"[Title/Abstract]
  OR chrononutrition[Title/Abstract]
  OR "time-restricted eating"[Title/Abstract]
  OR "time restricted feeding"[Title/Abstract]
)
AND
(
  inflammation[Title/Abstract]
  OR "oxidative stress"[Title/Abstract]
  OR "endothelial dysfunction"[Title/Abstract]
  OR "insulin resistance"[Title/Abstract]
  OR "gut microbiota"[Title/Abstract]
  OR microbiome[Title/Abstract]
  OR circadian[Title/Abstract]
  OR melatonin[Title/Abstract]
  OR orexin[Title/Abstract]
  OR leptin[Title/Abstract]
  OR ghrelin[Title/Abstract]
  OR tryptophan[Title/Abstract]
  OR "precision nutrition"[Title/Abstract]
```

```
  OR "personalized nutrition"[Title/Abstract]
  OR "individualized nutrition"[Title/Abstract]
  OR nutrigenomics[Title/Abstract]
  OR "GLP-1 receptor agonist*"[Title/Abstract]
  OR "dual incretin*"[Title/Abstract]
)
```

Search fields: MeSH terms and Title/Abstract.

Date range: January 2000 to June 2026.

Languages: English, Portuguese, and Spanish.

Database: PubMed/MEDLINE.

2. Scopus

Search strategy:

```
TITLE-ABS-KEY(
  "obstructive sleep apnea"
  OR "obstructive sleep apnoea"
  OR OSA
  OR "sleep-disordered breathing"
)
AND
TITLE-ABS-KEY(
  nutrition
  OR diet*
  OR "dietary pattern*"
  OR "nutrition therapy"
  OR "Mediterranean diet"
  OR "DASH diet"
  OR chrononutrition
  OR "time-restricted eating"
  OR "time restricted feeding"
)
AND
TITLE-ABS-KEY(
  inflammation
  OR "oxidative stress"
  OR "endothelial dysfunction"
  OR "insulin resistance"
  OR "gut microbiota"
  OR microbiome
  OR circadian
  OR melatonin
  OR orexin
  OR leptin
  OR ghrelin
  OR tryptophan
```

```
  OR "precision nutrition"
  OR "personalized nutrition"
  OR "individualized nutrition"
  OR nutrigenomics
  OR "GLP-1 receptor agonist*"
  OR "dual incretin*"
)
```

Search fields: Title, Abstract, and Keywords (TITLE-ABS-KEY).

Date range: January 2000 to June 2026.

Languages: English, Portuguese, and Spanish.

Database: Scopus.

3. Web of Science Core Collection

Search strategy:

```
TS=(
  "obstructive sleep apnea"
  OR "obstructive sleep apnoea"
  OR OSA
  OR "sleep-disordered breathing"
)
AND
TS=(
  nutrition
  OR diet*
  OR "dietary pattern*"
  OR "nutrition therapy"
  OR "Mediterranean diet"
  OR "DASH diet"
  OR chrononutrition
  OR "time-restricted eating"
  OR "time restricted feeding"
)
AND
TS=(
  inflammation
  OR "oxidative stress"
  OR "endothelial dysfunction"
  OR "insulin resistance"
  OR "gut microbiota"
  OR microbiome
  OR circadian
  OR melatonin
  OR orexin
  OR leptin
  OR ghrelin
```

```
  OR tryptophan
  OR "precision nutrition"
  OR "personalized nutrition"
  OR "individualized nutrition"
  OR nutrigenomics
  OR "GLP-1 receptor agonist*"
  OR "dual incretin*"
)
```

Search field: Topic (TS), including Title, Abstract, Author Keywords, and Keywords Plus®.

Date range: January 2000 to June 2026.

Languages: English, Portuguese, and Spanish.

Database: Web of Science Core Collection.

Additional search procedures

In addition to the electronic database searches, reference lists of key narrative reviews, systematic reviews, meta-analyses, and clinical guidelines were manually screened to identify potentially relevant publications not retrieved through the database searches.

The database searches were designed to maximize retrieval of literature addressing the relationship between nutrition and obstructive sleep apnea while also capturing mechanistic pathways, chronobiological factors, gut microbiota, and emerging precision-nutrition and incretin-based approaches.
